# Catchment landscape components alter relationships between discharge and stream water nutrient ratios in the Xitiao River Basin China

**DOI:** 10.1038/s41598-021-89804-1

**Published:** 2021-05-17

**Authors:** Changjun Gao, Wei Li, Lijuan Cui, Qiongfang Ma, Jian Cai

**Affiliations:** 1 Institute of Wetland Research, Chinese Academy of Forestry, Beijing Key Laboratory of Wetland Services and Restoration, No. 1, Dongxiaofu, Haidian District, Beijing, 100091 People’s Republic of China; 2 Guangdong Provincial Key Laboratory of Silviculture, Protection and Utilization, Guangdong Academy of Forestry, Guangzhou, 510520 People’s Republic of China; 3 Jilin Provincial Academy of Forestry Science, Changchun, 130033 People’s Republic of China

**Keywords:** Wetlands ecology, Environmental monitoring

## Abstract

The terrestrial environment of a watershed is a source of potential carbon (C), nitrogen (N), and phosphorus (P) exports, and the hydrological regime provides the mechanism to turn the potential exports into reality when water is available. However, the extent to which the terrestrial environment alters the strength and nature of streamflow in transporting stream water nutrient ratios remains largely unknown. This study combined monthly stream discharge data with synchronously sampled stream water C:N:P ratios in 14 catchment streams in the Xitiao River Basin (XRB) in Zhejiang Province, China. The transport effect of streamflow on C:N:P ratios varied depending on the nutrient element, flow condition, and terrestrial environment. In the lower reaches of the XRB, there were negative relationships between C:N ratios, C:P ratios and watershed discharge, and positive relationships between N:P ratios and watershed discharge in both high and low flow conditions. In the middle and upper reaches of the XRB, the C:N-discharge relationship changed from negative to positive when the streamflow conditions altered from low to high flow. The C:P- and N:P-discharge relationships were negative regardless of high or low flows, but the regression coefficient significantly decreased with increasing streamflow. The C:N-discharge correlation over the course of the year shifted from negative to positive, as urban areas expanded within the catchment. The C:P-discharge relationship altered from negative to positive with more cropland and wetland but from positive to negative with a greater forest percentage and mean percentage slope. Our results indicate that changes in the terrestrial environment (e.g., the proportion of a particular land cover within a watershed) generally produced a threshold flow above which the coupling relationships between element fluxes from the terrestrial to riverine ecosystem changed sharply.

## Introduction

As global climate change intensifies, extreme climate events (e.g., rainfall, drought and high temperatures) show an increasing trend. These climate events can affect the spatiotemporal pattern of rainfall intensity and frequency from the local to global scale^1^, and will potentially alter rainfall-runoff processes at a watershed-scale^2,3^. Watershed nutrient exports (e.g., carbon (C), nitrogen (N), phosphorus (P)), driven by rainfall-runoff, may become excessive compared with those in normal rainfall events over past decades^4,5^. The stoichiometric ratio (e.g., C:N:P ratio) is closely related to the C, N and P content and can affect the balance of elements exported into downstream ecosystems. It is regarded as a sensitive indicator affecting the trophic levels of downstream water at broad scales. Consequently, anomalous C:N:P ratios, owing to excessive C, N or P exports, could result in environmental problems and deleterious ecological effects^4,6,7^, such as downstream water eutrophication, alterations in trophic web structures and changing productivity of riverine ecosystems^8–10^. Previous studies have reported on the effects of instantaneous responses of nutrient ratio exports to extreme rainfall events and the long-term impacts of extreme rainfall events on nutrient ratio cycling on a time scale of decades^4,11^. This study contributes by studying these effects on the scale of a hydrological year.

Rainstorms, especially after a long drought, usually lead to a strong first-flush effect for nutrients in stormwater runoff from catchments. This first flush carries a large amount of C, N and P both from nutrient-rich catchment topsoil and from riparian floodplain subsoil into streamflow^6,12^. During dry seasons with few or no rain events, C, N and P may be primarily transported by subsurface runoff from multiple soil strata within the watershed. However, during rainy seasons with frequent rainstorms, these three nutrients may be exported mainly by overland flow in some areas and by subsurface discharge from deeper soil strata over the whole watershed^7,13^. The increase in extreme weather events will cause variations in the quantity and flow pathway of streamflow and will trigger differences in the quantity and form of nutrient ratios from season to season. For example, a substantial portion of dissolved phosphorus export appeared in stormflow while most nitrate nitrogen export occurred in non-stormflow within the Susquehanna River basin during a 50-year return period^13^. The quality (C:N) of large particles from the whole watershed did not change with flow condition, but that of smaller particles increased when particles flowed into a stream from the adjacent floodplain of the Ichawaynochaway Creek, USA during high streamflow periods^14^. The dissolved inorganic nitrogen:dissolved inorganic phosphorus ratios from the upper reaches of the Atchafalaya River to the lower Fourleague Bay changed from 54:1 to 32:1 during the spring peak discharge period^15^. Increases of rainfall intensity raised C, N, and P concentrations and loads during rainstorms within a purple soil watershed in China’s Sichuan Province^4^. Extreme rainfall events also significantly enhanced C:N and C:P ratios and reduced dissolved total nitrogen:dissolved total phosphorus ratios in rainstorm runoff compared with base flow.

It is known that rainstorm runoff will lead to more export of C, N and P in various forms compared with no-storm periods. However, the results on which source of nutrients have a greater output of C, N and P are not always consistent^4,16,17^. This suggests that other environmental drivers mediate the watershed export of C, N and P, such as physical geography, geology, land use, and the biogeochemical environment^7,18,19^. For example, streams draining forest land led to relatively more inorganic and total dissolved P, and a reduced N:P ratio compared with pasture in the western Amazon^20^. Streams draining wetland exported relatively larger particulate organic carbon (POC), and enhanced the seston C:P ratio compared with streams draining agricultural land in the Upper Peninsula of Michigan, USA^16^. The terrestrial environment in a watershed supplies the substrate of C, N or P export, which will potentially be exported through different transport pathways (e.g., subsurface or surface flow) from terrestrial to riverine systems when water is available^21,22^. However, the extent to which the terrestrial environment may alter the strength and nature of streamflow in transporting stream water nutrient ratios remains largely unknown.

We aimed to examine whether the relationships between stream discharge and stream water C:N:P ratios were mediated by different catchment landscape characteristics. In this paper, we measured the total nitrogen (TN), total phosphorus (TP) and total organic carbon (TOC) concentrations and stream discharges in 14 streams of the Xitiao River basin (XRB), Zhejiang Province, China on 12 sampling dates from July 2011 to June 2012. We first characterized the spatial–temporal variation in TN, TP and TOC concentrations and C:N:P ratios in two catchment feature clusters and in high and low flow conditions to investigate the impacts of catchment rainfall-runoff processes on nutrient export in different flow periods. We then examined whether these effects were altered by different landscape compositions among catchments. We predicted that different catchment landscape components would alter the relationships between C:N:P ratios and stream discharge, because different catchment landscape changes (e.g., expansion of agriculture and urbanization) alter the substrate supply of element nutrients which causes variations in the relative load and/or form of C, N and P with varying flow levels.

## Results

### Characteristics of catchment landscapes and groups

Catchment landscape compositions in the XRB streams are shown in Table 1. The three major landscape components in the XRB were forest (63.48%), cropland (24.24%) and urban land (7.58%). The remaining landscape types accounted for 4.7% of the XRB area. The landscape composition in the 14 sampled streams showed obvious differences. Nine landscape variables derived from remote sensing data and a digital elevation model (DEM) and also showed significant variations among the study streams in the XRB.

**Table 1:** Summary statistics for catchment landscape composition in 14 Xitiao River Basin stream.

Catchment	Size	% of each catchment area					
		Grass	Urban	Crop	Forest	Bare	Wetland
X1	96.56	2.07	5.62	66.18	15.15	4.24	6.53
X2	23.59	0.51	2.95	57.35	35.42	0.79	2.81
X3	148.63	0.21	6.15	30.99	60.92	0.14	1.59
X4	140.02	1.16	9.06	44.91	39.85	0.17	4.85
X5	55.81	0.45	9.70	67.39	20.15	0.05	2.08
X6	150.73	0.23	3.08	34.42	60.45	0.51	1.23
X7	147.75	0.12	11.15	10.55	77.07	0.00	1.11
X8	36.83	1.04	36.22	16.83	43.91	0.00	2.00
X9	52.48	0.11	4.55	10.60	84.71	0.00	0.03
X10	83.39	0.31	7.94	8.92	81.56	0.00	1.27
X11	95.05	0.41	11.02	19.55	67.93	0.44	0.64
X12	128.40	0.11	7.54	20.02	71.40	0.00	0.92
X13	355.18	0.14	1.37	11.09	85.17	0.00	2.13
X14	91.37	1.03	1.09	5.19	92.02	0.00	0.60

Size unit: km^2^.

Analysis of landscape composition clusters among the catchments in the XRB distinguished two different physiographic conditions (Table 2). Catchment group A consisted of X1, X2, X4, X5, and X8, while group B consisted of X3, X6, X7, X9, X10, X11, X12, X13, and X14. Six landscape variables showed significant variances between catchment groups A and B, e.g., percentage of grassland, percentage of cropland, percentage of forest, percentage of wetland, mean percentage of slope, and stream density (Table 2). Catchment group A represented the lower reaches of the XRB with relatively low topography, a more complex stream network, a greater proportion of wetland and cropland, and less forest compared with catchment group B in the middle and upper reaches.

**Table 2:** ANOVA of the differences of landscape variables between two catchment groups.

Landscape variables	Cluster		Error		F	Sig
	Mean square	df	Mean square	df		
Percentage of grassland	0.007	1	0.001	12	11.465	0.005
Percentage of urban	0.033	1	0.016	12	2.069	0.176
Percentage of cropland	0.458	1	0.029	12	15.986	0.002
Percentage of forest	0.752	1	0.019	12	39.843	0.000
Percentage of bareland	0.009	1	0.003	12	3.029	0.107
Percentage of wetland	0.026	1	0.002	12	14.734	0.002
Mean percentage of slope	0.141	1	0.003	12	40.988	0.000
Stream length	0.143	1	0.115	12	1.240	0.287
Stream density	0.360	1	0.074	12	4.892	0.047

Catchment group A (X1, X2, X4, X5, X8), and catchment group B (X3, X6, X7, X9, X10, X11, X12, X13, X14).

### Variations in nutrient concentrations and ratios

**Figure 1:**
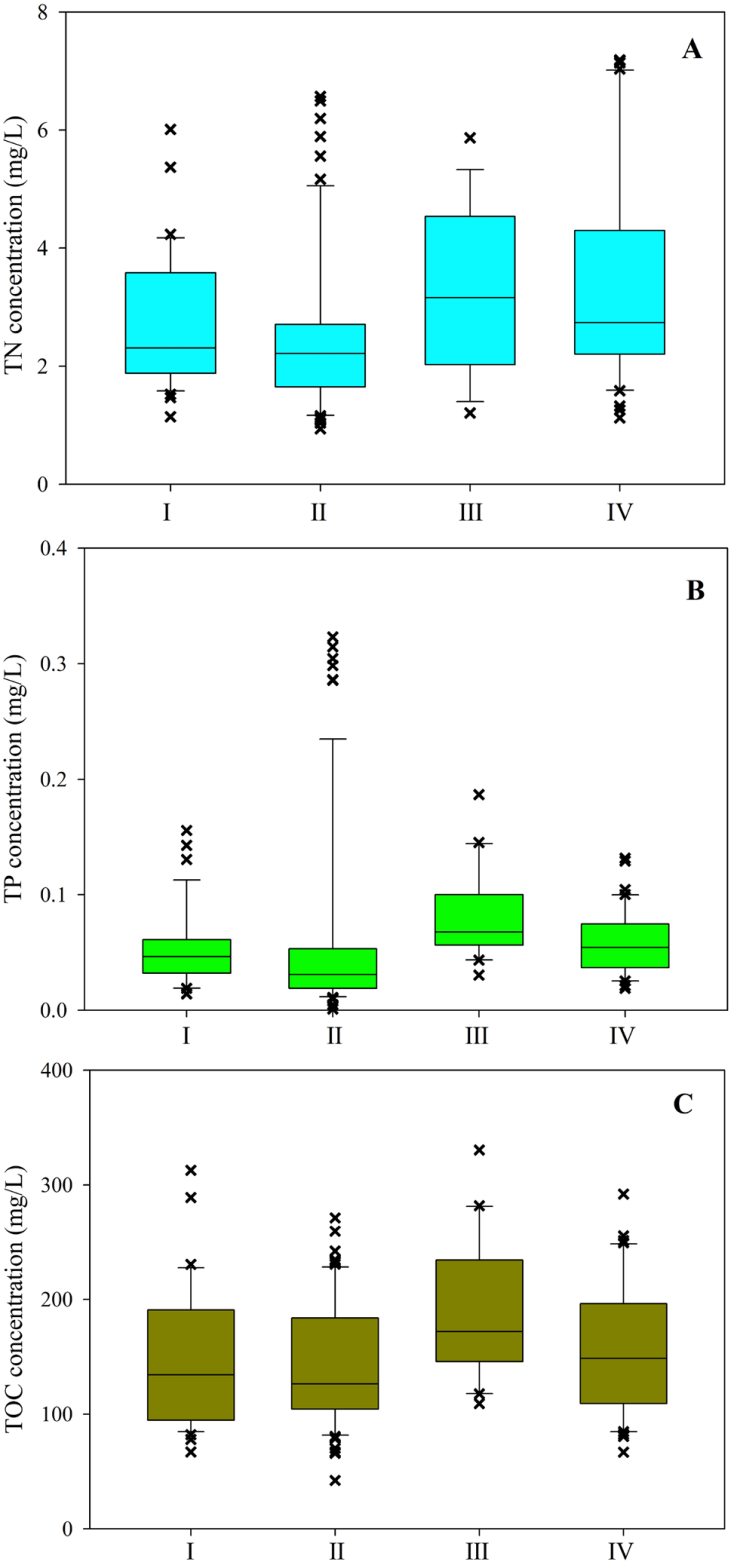
Box plots showing changes in TN, TP and TOC concentrations in the XRB during high and low flow periods. Note: I-The middle and upper reaches during high flow periods; II-The middle and upper reaches during low flow periods; III-The lower reaches during high flow periods: IV-The lower reaches during low flow periods.

The average nutrient concentrations in the XRB stream water during the study period were 2.9 ± 1.5 mg/L (TN), 0.067 ± 0.067 mg/L (TP), and 155.5 ± 63.3 mg/L (TOC) (Fig. 1). As shown in Fig. 1, all three nutrient concentrations demonstrated obvious variations in different locations of the XRB during high and low flow periods. The TN concentrations in the middle and upper reaches were generally lower than those in the lower reaches during the year. For example, TN concentrations in the middle and upper reaches varied from 1.1 mg/L to 5.1 mg/L, whereas TN concentrations in the lower reaches varied from 1.4 mg/L to 7.0 mg/L during the year (Fig. 1A). Generally, variations of TP and TOC concentrations both in different reaches of the XRB and during different streamflow conditions were similar to those of TN (Fig. 1B and C). The TP concentrations in the middle and upper reaches varied from 0.012 mg/L to 0.235 mg/L, while in the lower reaches they varied from 0.026 mg/L to 0.144 mg/L during the study. The TOC concentrations in the middle and upper reaches fluctuated between 82.07 mg/L and 228.51 mg/L and in the lower reaches they fluctuated between 85.58 mg/L and 282.02 mg/L during the year. On the whole, the three nutrient concentrations in the upper, middle and lower reaches during the high flow periods were slightly larger than those during the low flow periods.

As shown in Table 3 and Figure A.1. (see the Appendix), all three nutrient ratios showed considerable spatiotemporal variations across sampling sites, both among streams and within most streams. The average nutrient ratios in the XRB stream water during the study period were 67.3 ± 45.1 (C:N), 2782.8 ± 1304.4 (C:P), and 65.9 ± 47.4 (N:P). Generally, there was less N and P, and more C in stream water compared with Taihu Lake as indicated by high C:N and C:P ratios in stream water (Table 3). When averaged across the study period by stream, the C:N ratios were significantly related to both TOC concentrations (R = 0.59, *P* < 0.01) and TN concentrations (R = 0.77, *P* < 0.01). The C:P ratios were negatively correlated with TP concentrations (R = 0.83, *P* < 0.01) but were not significantly related to TOC concentrations (R = 0.1, *P* = 0.23). The N:P ratios were negatively correlated with TP concentrations (R = 0.8, *P* < 0.01) but were not significantly related to TN concentrations (R = 0.04, *P* = 0.59). The ANOVA results in Table 4 showed that 12.91%–29.23% and 30.06%–40.20% of variations in the C:N, C:P and N:P ratios were explained by stream sampling sites and sampling dates, respectively.

**Table 3:** summary statistics for C:N, C:P and N:P ratios in 14 Xitiao River Basin streams and Taihu lake.

Nutrient concentration and ratio	No. samples	Mean ± SD	CV	Min–Max	Source
**The XRB streams**					
TOC	168.0	155.5 ± 63.3			This study
TN	168.0	2.9 ± 1.5			This study
TP	168.0	0.067 ± 0.067			This study
C:N	168.0	67.3 ± 45.1	67.0	14.8–252.2	This study
C:P	168.0	2782.8 ± 1304.4	46.9	492.1–5763.9	This study
N:P	168.0	65.9 ± 47.4	71.9	8.8–276.0	This study
**The Taihu Lake**					
TOC		27.9 ± 2.35			Hu et al.^29^
TN	72.0	3.45		1.24–9.48	Xu et al.^26^
TP	72.0	0.15		0.08–0.32	Xu et al.^26^
C:N	14.0	6.11 ± 1.54	25.16	4.14–9.24	Qu et al.^27^
C:P	14.0	20.57 ± 10.93	53.15	11.53–47.12	Qu et al.^27^
N:P	660.0	65.6 ± 49.3	75.2	5.2–126.0	Liu et al.^28^
	72.0	39.5 ± 26.7	67.6	15.0–80.0	Xu et al.^26^
**The Yukon River**^*a*^					
C:N		31.58 ± 8.86		21.45–46.25	Guo et al.^31^
C:P		8905.91 ± 4806.04		4660.5–15,942.9	Guo et al.^31^
N:P		320.67 ± 220.83		122.06–743.14	Guo et al.^31^
**The Yangzte River**^*b*^					
C:N		11.08 ± 1.39		8.6–13.67	Gao et al.^30^
C:P		102.82 ± 43.14		56.67–172.22	Gao et al.^30^
N:P		9.72 ± 5.08		4.0–20.0	Gao et al.^30^

Nutrient concentration units is mg/L. ^a^C, N and P means dissolved organic carbon (DOC), dissolved total nitrogen (DTN) and dissolved total phosphorus (DTP). ^b^C, N, P means particulate organic carbon (POC), particulate nitrogen (PN) and phosphate (PO_4_^3−^).

**Table 4:** ANOVA of the effects of sampling site and date on C:N, C:P, and N:P ratios in Xitiao River Basin streams.

Source	df	C:N			C:P			N:P		
		*F*	*P*	% var	*F*	*P*	% var	*F*	*P*	% var
Model	24	8.57	< 0.01	59.32	6.24	< 0.01	51.33	8.89	< 0.01	61.43
Date	11	9.23	< 0.01	29.23	3.42	< 0.01	12.91	5.79	< 0.01	18.34
Site	13	8.01	< 0.01	30.06	8.61	< 0.01	38.34	10.75	< 0.01	40.20
Error				40.68			48.67			38.57

Percentage of variance (% var) was calculated as sum of squares of treatment / total sum of squares.

### Relationships between nutrient ratios and discharge in high and low flow periods

**Figure 2:**
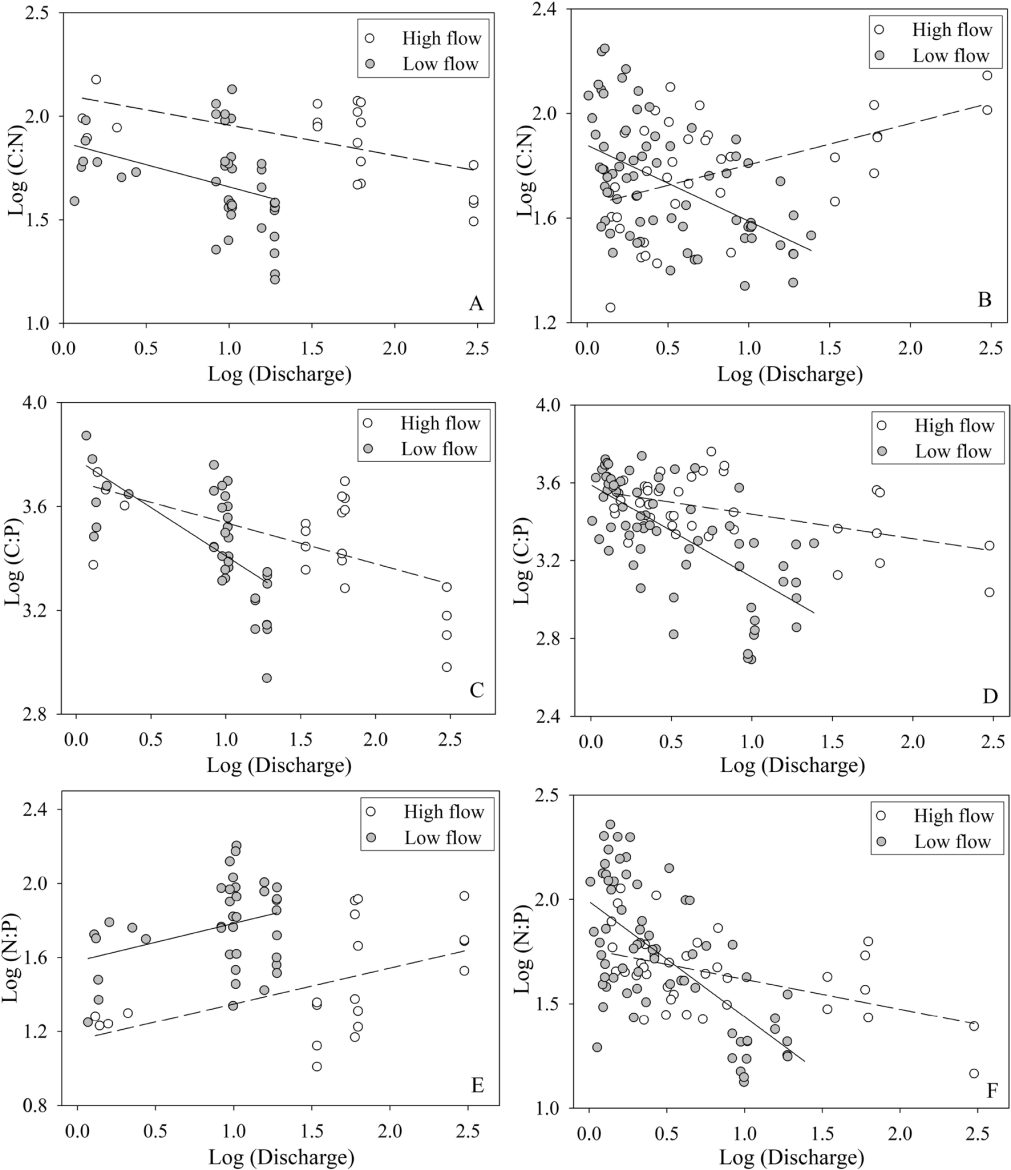
Linear regressions between C:N:P ratios and discharge during periods of high and low flow. Notes: Panels A, C, & E pertain to group A while B, D, &F pertain to group B. Group A consist of X1, X2, X4, X5, X8 while group B consist of X3, X6, X7, X9, X10, X11, X12, X13, X14. Discharge data and C:N:P ratios derived from the fourteen sampling streams from X1 to X14 across the study period.

The C:N ratios in catchment group A were negatively correlated with discharge during high and low flow conditions (y =  − 0.147x + 2.104, R^2^ = 0.35, *P* = 0.006, n = 20; y =  − 0.215x + 1.874, R^2^ = 0.136, *P* = 0.019, n = 40, respectively; Fig. 2), and were not significantly correlated to discharge during the whole sampling period (*P* = 0.281, data not shown). The C:N ratios in catchment group B increased with increasing discharge during high flow conditions (y = 0.158x + 1.646, R^2^ = 0.23, *P* = 0.003, n = 36), decreased with increasing discharge during low flow conditions (y =  − 0.291x + 1.879, R^2^ = 0.27, *P* = 0.000, n = 72), and did not vary with discharge during the whole sampling period (*P* = 0.554, data not shown). In contrast, the C:P ratios decreased significantly with increasing discharge during all flow conditions, and in both catchment groups. For example, the C:P ratios in catchment group A were negatively correlated with discharge during high and low flow conditions (y =  − 0.158x + 3.695, R^2^ = 0.35, *P* = 0.007, n = 20; y =  − 0.372x + 3872, R^2^ = 0.44, *P* = 0.000, n = 40, respectively; Fig. 2), and over the whole sampling period (*P* = 0.000, data not shown). The C:P ratios in catchment group B showed similar and significant trends to those in catchment group A during high, low, and whole flow conditions (*P* = 0.002, *P* = 0.000, *P* = 0.000, respectively; Fig. 2). The N:P ratios in catchment group A were positively correlated with discharge in high and low flow conditions (y = 0.194x + 1.155, R^2^ = 0.27, *P* = 0.019, n = 20; y = 0.201x + 1.581, R^2^ = 0.11, *P* = 0.038, n = 38; respectively; Fig. 2), and were not significantly correlated to discharge in the whole study period (*P* = 0.637, data not shown). Conversely, the N:P ratios in catchment group B decreased significantly with increasing discharge in high and low flow conditions, and the whole study period (y =  − 0.144x + 1.761, R^2^ = 0.25, *P* = 0.002, n = 36; y =  − 0.552x + 1.990, R^2^ = 0.43, *P* = 0.000, n = 69; y =  − 0.307x + 1.880, R^2^ = 0.30, *P* = 0.000, n = 105; respectively; Fig. 2).

### Effects of streamflow on nutrient ratios

**Figure 3:**
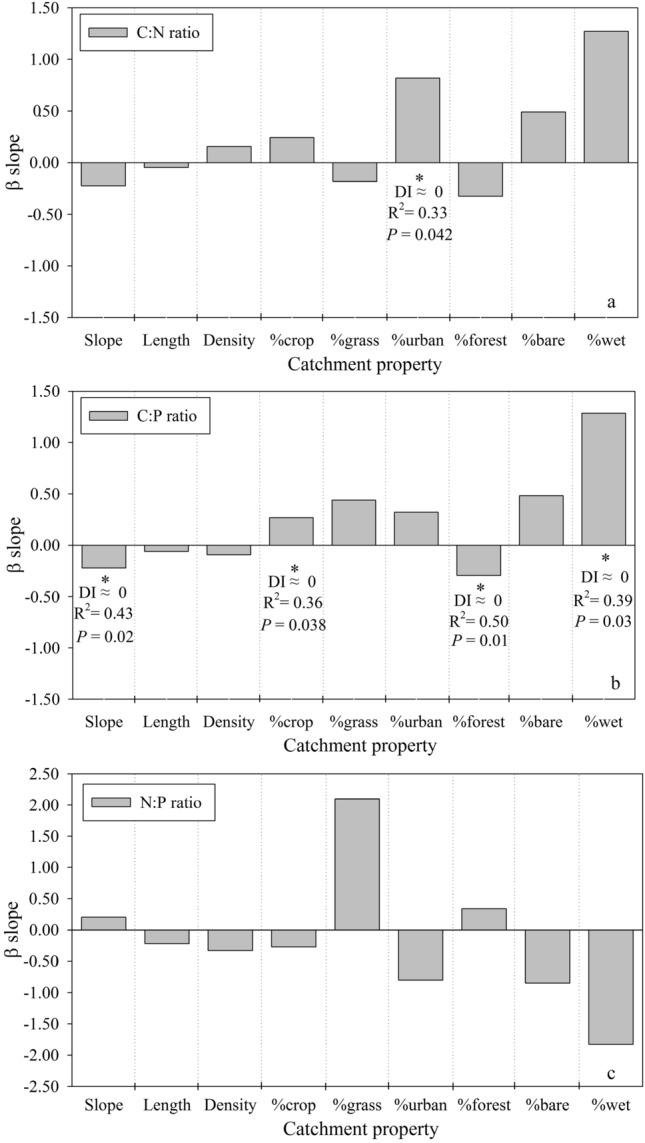
β slopes for relationships between each catchment characteristic and α slopes each element ratio (C:N, C:P, and N:P) and stream discharge. An asterisk indicates that the β slopes was found to be significantly different (*P* < 0.05) from zero. For significant β slopes, we indicate whether the difference index (DI) was positive, negative or directional and show the R^2^ of the relationships.

Figure 2 showed that there were significant differences for C:N:P ratios both among the catchment groups and during different flow periods. To examine whether different landscape compositions in the XRB catchments could explain the variations in the relationships between nutrient ratios and stream discharge among sampling sites, a directional index (DI) was developed to quantify the impacts of catchment properties on the stream-specific relationships between stream water C:N:P ratios and discharge. Detailed information on calculating the DI can be found in the Appendix. The DI results for the mediated effects of the catchment terrestrial environment on the nutrient ratio-discharge relationships are shown in Fig. 3. Several landscape compositions explained 57%–71% α of the variability in the relationships between stream water C:N and C:P ratios and discharges (Fig. 3). Figure 3 also illustrates that the direction of the relationships between stream water C:N and C:P ratios and discharge were significantly altered, but the strength of the C:N and C:P ratio-discharge relationships (directional relationship/DI ≈ 0, Fig. 3) were almost unchanged, under different landscape compositions in the 14 catchments. The percentage of urban area was the sole significant predictor for the C:N-discharge relationships. With increases in the percentage of urban land in some catchments, the direction of the C:N ratio-discharge relationships changed from a negative correlation into a positive correlation (Fig. 3a). The direction of the relationships between stream water C:P ratios and discharge also changed significantly, with variations in four landscape components (e.g., mean percentage of slope, percentage of cropland, percentage of wetland and percentage of forest, Fig. 3b). The C:P ratio-discharge relationships altered from negative to positive with more cropland and wetland. In contrast, the C:P ratio-discharge relationships changed from positive to negative when the percentage of forest and the mean percentage slope increased (Fig. 3b). The strength and direction of relationships between stream water N:P ratios and discharge did not vary significantly with changing landscape compositions in the studied catchments (Fig. 3c).

## Discussion

Nine catchment landscape variables (Table 5) were selected from previous landscape studies of nutrient concentrations or load in the same streams of the Xitiao River catchment, China^23,24^ and from previous similar studies^16,25^. Although several of the catchment properties were significantly correlated with one another (see Appendix Table A.1.), the full set of catchment properties was retained because of their individual potential to account for differences in the nutrient budgets of stream ecosystems. Analysis of clustering in landscape characteristics among catchments in the XRB distinguished two different physiographic conditions (Table 2). Catchment groups A and B not only represented differences in the landscape structure but also in the topographical conditions. In addition, the soils in catchment groups A and B were dominated by paddy soil and red soil, respectively. Under the two different physiographic conditions, streamflow had a different effect on stream water nutrient concentrations and ratios during high and low flow conditions.

**Table 5:** Descriptive statistics of landscape variables of the study streams in the Xitiao River Basin.

Landscape variable	Abbreviation	Units	Mean	SD	Max	Min
Mean percentage of slope	Slope	%	14.03	7.57	27.72	2.59
Stream length	Length	km	21.21	11.24	43.81	8.05
Stream density	Density	km^−1^	0.21	0.07	0.34	0.11
Percentage of cropland	%crop	%	28.86	21.90	67.39	5.19
Percentage of grassland	%grass	%	0.56	0.57	2.07	0.11
Percentage of urban	%urban	%	8.39	8.68	36.22	1.09
Percentage of forest	%forest	%	59.69	24.94	92.02	15.15
Percentage of bareland	%bare	%	0.45	1.12	4.24	0.00
Percentage of wetland	%wet	%	1.98	1.76	6.53	0.03

From July 2011 to June 2012, the average nutrient concentrations in the XRB stream water were obviously higher for TOC concentration and lower for TN and TP concentrations when compared to those in Taihu Lake (Table 3). Likewise, the monthly nutrient ratios (C:N, C:P, and N:P) in the 14 sampling streams in the XRB during the studied period were significantly higher than those in both the surface sediments and water column of Taihu Lake in earlier studies^26–29^. Compared with other large rivers of the world, nutrient ratios in the XRB also have a relatively higher value of C:N ratio and a relatively moderate value of C:P and N:P ratios^30,31^ (Table 3). Limestone mining in upland of the XRB caused exposed red latosol soil, and mineral and sand shipping and washing in streams led to large volumes of particulate matter in surface river water and resuspension of river sediments. These anthropogenic activities led to relatively more C and less N and P in stream water of the XRB when compared to the downstream freshwater (e.g., the Taihu Lake) and other large rivers of the world (e.g., the Yangtze River and the Yukon River). Similar results can be found in the Biscayne Bay, Florida^32^. In addition, the element ratios were also significantly larger (C:N:P molar ratio, 4620:70:1 in this study) than the Redfield ratio (C:N:P molar ratio, 106:16:1)^33^, suggesting differences in the primary drivers for nutrient transport processes in lotic ecosystems, and in lake and/or marine ecosystems.

Visible changes in C:N:P ratios resulted in different catchment terrestrial source^4^. For example, the DOM and POM sources shifted from mostly terrestrial during seasonal freeze–thaw cycles (e.g., winter and spring) to a mixed aquatic-terrestrial DOM and POM during the ice-free season (e.g., plum rain season and heavy rainfall)^34^. During the ice-free season, rainfall may enhance terrestrial inputs but retard aquatic production of DOM and POM, while the no-rainfall or low-rainfall months may lead to a considerable aquatic contribution to DOM and POM^34^. In addition, the C:N:P ratios were strongly affected by the P fraction in surface-adsorbed and intracellular P pools, soil N leaching and aquatic organisms absorbing or releasing N^4,35,36^. The nutrient concentrations and ratio flows for each stream through time showed there was a relatively tight coupling of C, N and P in these streams suggesting similar or transmission pathways for nutrients from uplands to streams in the XRB. The results of ANOVA analysis (Table 4) showed that 12.91%–29.23% and 30.06%–40.20% of the variation in C:N, C:P, and N:P ratios was explained by stream sampling sites and sampling dates, respectively. Consequently, the variability in C:N, C:P, and N:P ratios in the XRB stream water reflected environmental processes that vary through time (e.g., seasonal fluctuations in stream discharge) and features that vary across space (e.g., landscape properties that varied among catchments)^16^. Previous studies have reported similar results^37,38^. About 30.57% of the variation in C:N:P ratios could not be explained by the catchment environmental features and processes selected in this study. Two reasons may explain this phenomenon. First, base flow accounts for 27% of the streamflow in the XRB^39^, and this may have an important impact on a fraction of the nutrient concentration, regardless of the catchment landscape component^40^. However, this study could not distinguish the proportion of base flow from streamflow in the XRB. Second, point source pollution discharge in the XRB may contribute to a small fraction of the C:N:P ratio variations, because of uneven distribution of some discharge outlets of industrial wastewater and municipal sewage.

C:N ratios generally decreased with increasing discharge in the XRB (Fig. 2). This was associated with the way in which stream discharge temporally increased N export during rainstorms and depleted C availability under base flows. During high flow conditions, the C:N ratios in streams were derived mainly from terrestrial DOM and POM, which were primarily triggered by heavy rainstorms or runoff processes^34,41^. However, the C:N ratios in upland soils were larger than those in lowland soils. Therefore, the upland soil DOM and POM with higher C:N ratios were transported to streams during high flow conditions. This explains why the C:N ratios in catchment group B maintained a positive relationship with discharge during high flow conditions. The same reasons can explain the negative relationships between C:P ratios and discharge. For example, significant P leaching and release were observed with rainstorms, and particulate and dissolved C export was lower and depleted by aquatic organisms under base flow conditions and runoff during low rainfall.

Catchment group A mainly represented the lower reaches of the XRB. In these regions, the N:P ratios increased with discharge under both base flows (e.g., low flow conditions) and during heavy rainfall or runoff processes (e.g., high flow conditions). This was explained by the fact that the increase in N leaching during extreme rainfall was greater than P losses during erosion^4^. P is also absorbed swiftly by soil particulates and desorbed slowly and competes with dissolved organic carbon (DOC) sorption–desorption during rainfall or runoff processes^42^. Therefore, these rainfall-runoff processes led to more N release than P release. There were more than 30 dams in the lower reaches of the XRB which could cause large amount of P retention in sediments compared to N when they move from upstream to downstream during base flows or low rainfall periods. This was also the reason why N:P ratios in low flow periods were higher than that in high flow periods. Conversely, the N:P ratios showed a decreased trend with discharge in the middle and upper reaches of the XRB (e.g., catchment group B) during low and high flow periods. This can be explained primarily by differences in landscape and anthropogenic elements in catchment group B compared with catchment group A. Forest accounted for 60.92–92.02% in catchments of the middle and upper reaches of the XRB (Table 1) and which was dominated by artificial moso bamboo plantation. The red latosol soil in subtropical China was usually P-limited. So, excessive phosphorus fertilizer was often applied in local bamboo plantation to increase the production of bamboo wood and bamboo shoots. This consequently contributed to a relatively more P leaching than N leaching and caused the negative relationship between N:P ratios and discharge in catchment group B.

Runoff processes from the upper catchment surface soil or deeper soil to downstream rivers play a critical role in transporting nutrients. The dynamics of the C, N and P element ratios were inherently linked to streamflow, and their linkage was temporal and spatially variable because of different landscape composition (e.g., land cover, soil type)^43–45^. Consequently, these landscape-mediated variations in the relationships between discharge and C:N:P ratios may be explained by the determination of a threshold flow, at which the balance of the element flux changes owing to different landscape compositions among catchments.

As shown in Fig. 3a,b,c (and the Appendix), landscape variables generally influenced the direction of the relationships between stream discharge and nutrient ratios, with the exception of N:P ratios. The relationships between discharge and C:N ratios shifted from negative to positive as the percentage of urban land among catchments increased, which suggests that the relative loads and sources of C and N in urban streams would be reversed as urban areas expand within a catchment. During the low flow periods, point source pollution (e.g., wastewater treatment plant), indicated by the small percentage of urban area within a catchment, could play critical role in N rather than C loading. As the expansion of urban area, urban land (e.g., mining and impervious surface landscape) replacing point source pollution became the main source of C export during the high flow periods. Sources of nutrient loading from watersheds may be changed largely either in individual storms in the same region or in the same storm in different region^30^. Besides, the impact of landscape characteristics in surrounding catchments could play threshold effects on stream chemical and biological indicators^46,47^. For example, declines in fish fauna condition in Wisconsin streams occurred only after 20% of the catchment was urbanized or agriculture occupied 50% of the catchment^48^. This may be attributed to human alterations of terrestrial landscapes, e.g., urbanization, which could lead to greater primary productivity in urbanized streams than in pristine streams^49^. Agricultural and urban land use types are considered to result in significant morphological alterations and pressures in freshwater ecosystems when they exceed 40% and 15% of the catchment area, respectively^50^. Similar drivers can be used to explain shifts in the directions of the effects of several landscape indicators (e.g., slope, percentage of cropland, percentage of forest, and percentage of wetland) on the relationships between discharge and C:P ratios. None of the selected landscape variables had any influence on the relationships between discharge and N:P ratios (see Methods). In human-dominated catchments, the natural mechanisms of N and P export from artificial landscape types may be influenced by changes in flow confluence processes and varying infiltration capacities in a catchment^51^, which suggests that landscape effects on discharge and N:P ratio relationships may be uncertain. The C:P and N:P ratios were significantly correlated with TP concentration and were not related to TOC and TN concentration. However, the C:P-discharge relationships can be explained by certain landscape variables. This may indicate that some fraction of TOC may contribute to the C:P-discharge relationships and no fraction of the TN can explain the N:P-discharge relationships.

The XRB represents a typical watershed in eastern China, which is characterized by intensive agricultural activity, rapid urbanization, and periodic reforestation and deforestation. These anthropogenic elements are regarded as dominant terrestrial sources of non-point source pollution. Therefore, controlling nutrient exports from terrestrial to riverine ecosystems is crucial. The dynamics of C:N:P ratios in this study indicated that there was relatively higher C and N and less P in streams compared with the Redfield ratio, and that the C:N and C:P ratios generally decreased with stream discharge during different flow conditions resulting from different hydrological transport pathways. During high flow conditions (e.g., the rainy season), regulatory measures should focus on reducing terrestrial particulate C and N loads, e.g., reducing bamboo forest harvesting and fertilizer application, increasing plant cover along ditches and in catchments, and slowing down flow velocities by increasing ditch cambers and lengths^4,52^. During low flow conditions (e.g., the dry season), attention should be paid to modulating the balance of C and N in aquatic ecosystems e.g., increasing flow velocities and decreasing residence time by opening upland dams, sediment dredging, and rehabilitating emergent macrophytes in nearshore waters. Because the balance of element fluxes can be reversed by changing the percentage of a certain land cover in a catchment (e.g., urban, cropland or forest), watershed managers should understand that rational land use planning (e.g., eco-urbanization) and scientific agricultural and forestry management activities (e.g., eco-agriculture, selective cutting or shelterwood cutting) could reduce the cost of water pollution and improve aquatic ecosystem functionality at watershed scale.

## Conclusions

Streamflow in the upper catchment exported relatively more TOC than TN and TP, with relatively higher C:N and C:P ratios and roughly equal N:P ratios, over the course of a year, compared with the downstream XRB. There were highly variable relationships between nutrient ratios and streamflow under different catchment environments. In the lower reaches of the XRB, with characteristics of relatively flat topography, a more complex stream network, and a larger percentage of wetland and cropland, the transmission effects of streamflow on nutrient ratios were relatively stable without changing flow periods. In these regions, increased streamflow caused a greater C loss compared with N and P, and increased N leaching compared with P during the low and high flow periods. Conversely, in the middle and upper reaches of the XRB, enhanced streamflow increased C loss compared with N during the low flow period. The opposite trend was seen during the high flow period. For C:P and N:P ratios, increased streamflow led to a reduced loss of P compared to C and N during the low and high flow periods. However, the transmission effect, indicated by the regression coefficient, significantly decreased when the streamflow changed from low flow to high flow. Furthermore, the direction (e.g., positive or negative) of the C:N- and C:P-discharge relationships clearly changed with different landscape composition among catchments over the course of 1 year. This consequently indicated that changing the terrestrial environment (e.g., the proportion of a particular land cover within a watershed) generally produced a threshold flow above which the coupling relationships between element fluxes from terrestrial to riverine ecosystems changed sharply. The results from this study should help us understand the mechanisms and consequences of landscape change on nutrient transport in lotic ecosystems and may provide a theoretical basis for managers to minimize the adverse effects of catchment land use and hydrological changes on receiving streams.

## Material and methods

### Site description

**Figure 4:**
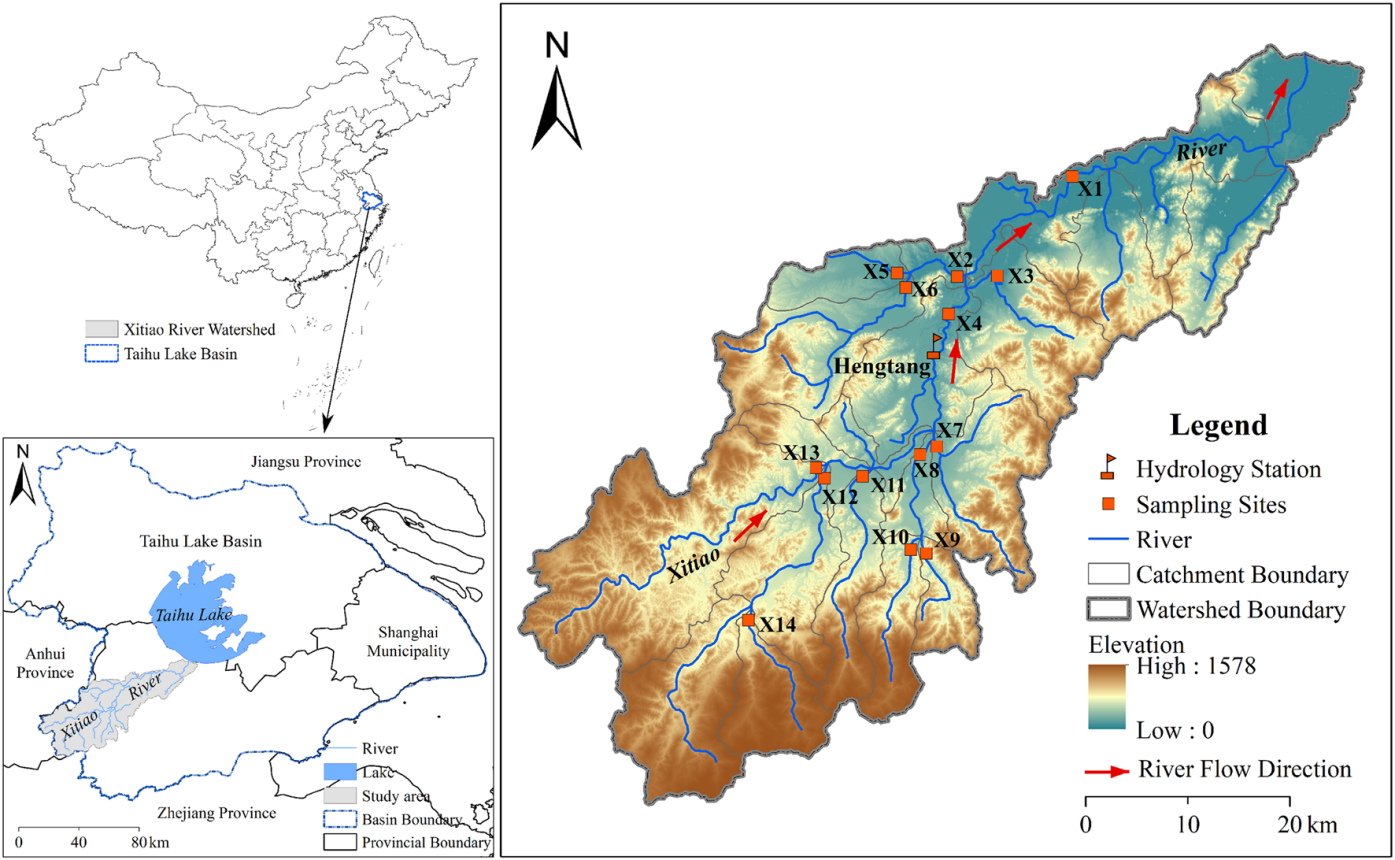
Map of the study area and surface water sampling sites. Note: names and coordinates for fourteen sampling sites are listed as follows, X1-Xiaoxikou (N 30° 52′ 3.77″, E 119° 52′ 8.20″), X2-Hunni port (N 30° 48′ 48.33″, E 119° 44′ 6.27″), X3-Kuntong port (N 30° 48′ 31.17″, E 119° 46′ 35.39″), X4-Hengtang village (N 30° 46′ 56.50″, E 119° 43′ 14.12″), X5-Nihe stream (N 30° 48′ 27.10″, E 119° 40′ 26.59″), X6-Shahe stream (N 30° 48′ 37.64″, E 119° 40′ 50.5″), X7-Dixi creek (N 30° 39′ 9.84″, E 119° 41′ 17.99″), X8-Huxi creek (N 30° 39′ 53.09″, E 119° 40′ 11.7″), X9-Gangkou part (N 30° 34′ 42.76″, E 119° 39′ 39.97″), X10-Shanhe port (N 30° 34′ 2.27″, E 119° 38′ 45.62″), X11-Longwangxi creek (N 30° 38′ 11.49″, E 119° 36′ 27.61″), X12Nanxi creek (N 30° 38′ 23.09″, E 119° 34′ 6.29″), X13-Xixi creek (N 30° 39′ 59.74″, E 119° 33′ 40.72″), X14-Luojiafei reservoir (N 30° 32′ 37.39″, E 119° 28′ 8.59″). Using ArcGIS10.4 (https://www.esri.com/zh-cn/arcgis/products) software to create the spatial datasets of the sampling sites.

The XRB (119° 14′–120° 29′ E, 30° 23′–31° 11′ N) is located in Zhejiang Province, China (Fig. 4). It falls within the Taihu Lake basin, the third largest freshwater lake in China. The main stem of the Xitiao River is about 145 km long, while the watershed area is 2274 km^2,23^. The Xitiao River is one of the most important inflow sources of water for Taihu Lake and has a mean annual discharge of 487 m^3^/s and a total river discharge of 15.4 × 10^9^ m^3^/year (Huzhou Water Resources Bureau, 2012; http://slj.huzhou.gov.cn.). Discharge in the Xitiao River is derived mainly from surface flow and near-surface flow, which is dominated by different landscape compositions within the catchment^39^. This area has a subtropical monsoon climate, and is characterized by a moderate and moist climate, abundant precipitation, and four distinct seasons. The annual average temperature varies from 15.5 °C to 15.8 °C. The annual average precipitation is 1460 mm, 75% of which occurs between May and September. Precipitation increases with altitude in this region. The altitude of the XRB from north to south ranges from 0 to 1578 m, while rainfall increases from 1333.5 mm to 1645.3 mm^23^. Extreme drought occurred in the spring of 2011 and was defined as a once in 60-year event. Extreme rainfall occurred in the summer of 2012 and was listed as a once in 50-year event (Zhejiang Meteorological Bureau, 2011; 2012; http://zj.weather.com.cn/qhbh/qhgb/index.shtml.). The XRB is a typical and representative watershed of the eastern plains of China.

**Figure 5:**
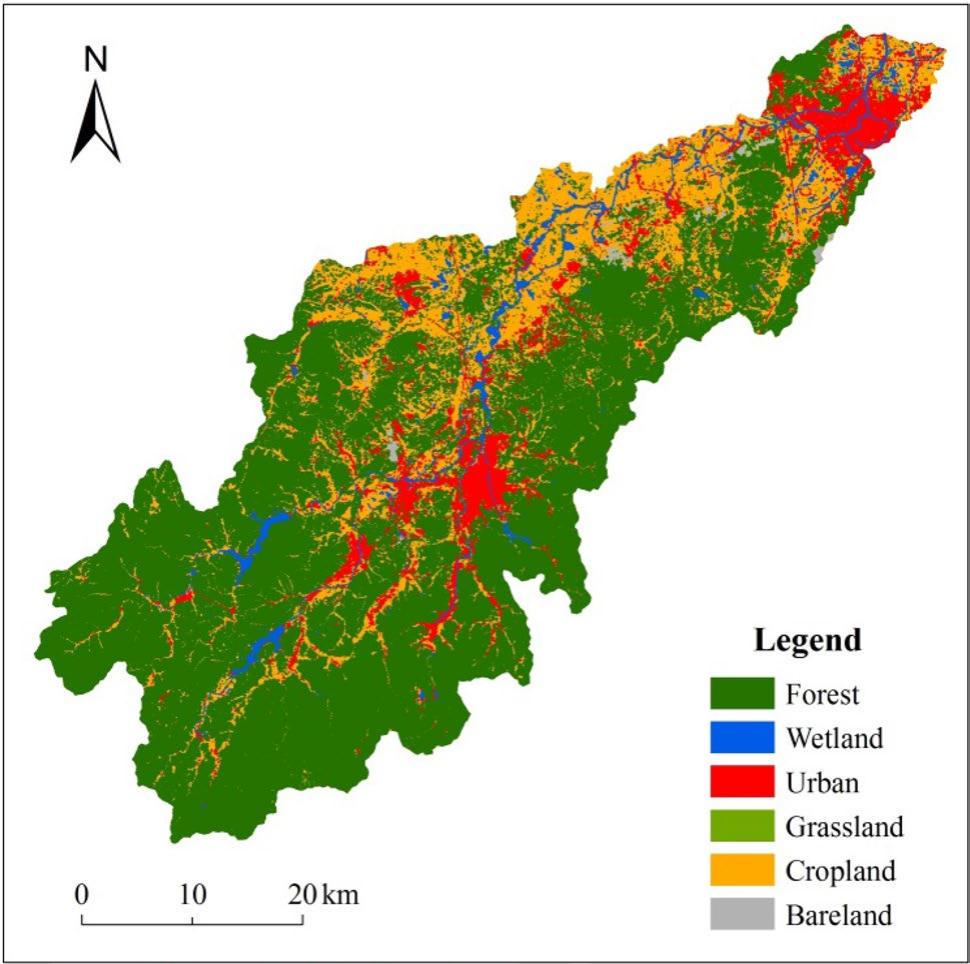
Landscape classification for the study area. Note: Using ArcGIS10.4 (https://www.esri.com/zh-cn/arcgis/products) software to create the spatial datasets of the landscape classification in the study area.

The XRB has a complex landscape composition, as well as varying geomorphology in its sub-basins. The upper reaches of the catchment are mountainous, and land use is dominated by forest. The middle reaches are hilly, and land is mainly used for paddy fields and urban development, while the lower reaches are characterized by plains, and substantial urban areas (Fig. 5). Land use in the XRB is made up of forest (63.48%), wetland (3.29%), urban land (7.58%), grassland (0.78%), cropland (24.24%) and bare land (0.63%) (Fig. 5).

### Sample collection and water chemical analysis

The 14 streams investigated in this study included small streams, headwater streams and the main stem of the Xitiao River (Fig. 4). Nested sites (sites located downstream of other sites) were chosen to ensure a wide and even geographical coverage of the XRB. Other researchers have used this sampling method successfully for analyzing the relationships between catchment landscape variables and stream dissolved or particulate nutrients^16,53,54^. Duplicate water samples were collected monthly from July 2011 to June 2012 from three points across the middle depth of each stream (left side, central channel, and right side); the water depth of the main channel in most of the streams sampled was less than 1.5 m. Samples from the three collection points were mixed in acid-washed 1000-mL polypropylene bottles at a ratio of 1:1:1, resulting in a sample that represented the whole water column. All water samples were stored in a portable incubator at 4 °C and transported to the laboratory to be processed. Samples for DOC and POC measurements were filtered through pre-ashed Whatman GF/F filters within 6 h of collection. The filtered water was used to measure the DOC concentration. The suspended particles collected from the pre-ashed Whatman GF/F filters were dried for 24 h at 60 °C and were subsequently fumigated with 38% HCl acid in a desiccator for 24 h and were used immediately for analysis of POC. The DOC concentrations were determined by a high-temperature combustion method using a total organic C analyzer (Shimadzu TOC-V), while POC concentrations were determined using a C/H/N/S/O_2_ analyzer (EA 3000 CHNS/O Analyzer). The TOC concentrations were calculated as the sum of DOC and POC. When all water samples were transported to the laboratory, they were immediately tested for TN and TP concentration by spectrophotometric analysis after a combined persulfate digestion^26,55^. In this study, we focused on the total fractions of organic carbon, nitrogen and phosphorus because they included the entire bioavailable pool; the non-bioavailable fractions may become bioavailable during longitudinal transport within river ecosystems^56,57^. Nutrient mass ratios (C:N, C:P, and N:P) were calculated from the TOC, TN, and TP measurements. Prior to all analyses, nutrient ratios were log_10_ transformed to improve the distribution and homogeneity of variance.

### Acquisition and analysis of watershed landscape properties

A cloud-free Landsat TM image (path/row: 119/39; date: 24 May, 2010), obtained from the Earth Resources Observation and Science Center (http://glovis.usgs.gov/), was used to map the landscape types of the XRB. Eighteen sections of relief maps of the study region (scale 1:50,000), purchased from the National Administration of Surveying, Mapping and Geoinformation (http://www.sbsm.gov.cn/), provided information on watershed topographic properties.

The image and all the relief maps were georectified to an Albers equivalent conical projection, at an accuracy of less than 0.5 pixels. Bands 1–5 and 7 of the satellite imagery were used for mapping landscape types. A supervised classification using the maximum likelihood method, combined with visual interpretation, was used to distinguish six landscape types: forest, wetland (e.g., reservoir, pond, and river), urban (e.g., town, rural settlement, and road), grassland, cropland (e.g., dry land and paddy field), and bare land (e.g., wasteland and abandoned land after mining) in ENVI 4.7 software. The accuracy of the remote sensing classification was assessed using a 2011–2012 ground truth survey and high-resolution images from Google Earth. The total accuracy of the landscape classification was above 85%, which was considered sufficient for this study. All the relief maps were digitized to generate a DEM of the study region (Fig. 4) at a resolution of 25 m in ArcGIS 10.4 software (https://www.esri.com/zh-cn/arcgis/products). The DEM data were used to generate catchment boundaries and to calculate the catchment characteristics (e.g., catchment area, mean percentage of slope) for each sampling point. The catchment boundaries were subsequently used to clip the landscape classification data, which were summarized into the following categories for each catchment: percentage of catchment area in forest, wetland, urban, grassland, cropland, and bare land. The stream length and stream density for each investigated catchment were also calculated. Summary statistics of catchment landscape properties indicated considerable variation among stream catchments for the XRB (Tables 1 and 5). For all subsequent statistical analyses, stream length, stream density, and mean catchment slope were log_10_ transformed and proportions of each landscape type in each catchment were arcsine-square root transformed to stabilize variances. Two catchment groups were identified to represent differences in landscape compositions in the 14 sample catchments using K-means clustering (Table 2), e.g., group A (X1, X2, X4, X5, and X8), and group B (X3, X6, X7, X9, X10, X11, X12, X13, and X14).

### Determination of stream discharge

**Figure 6:**
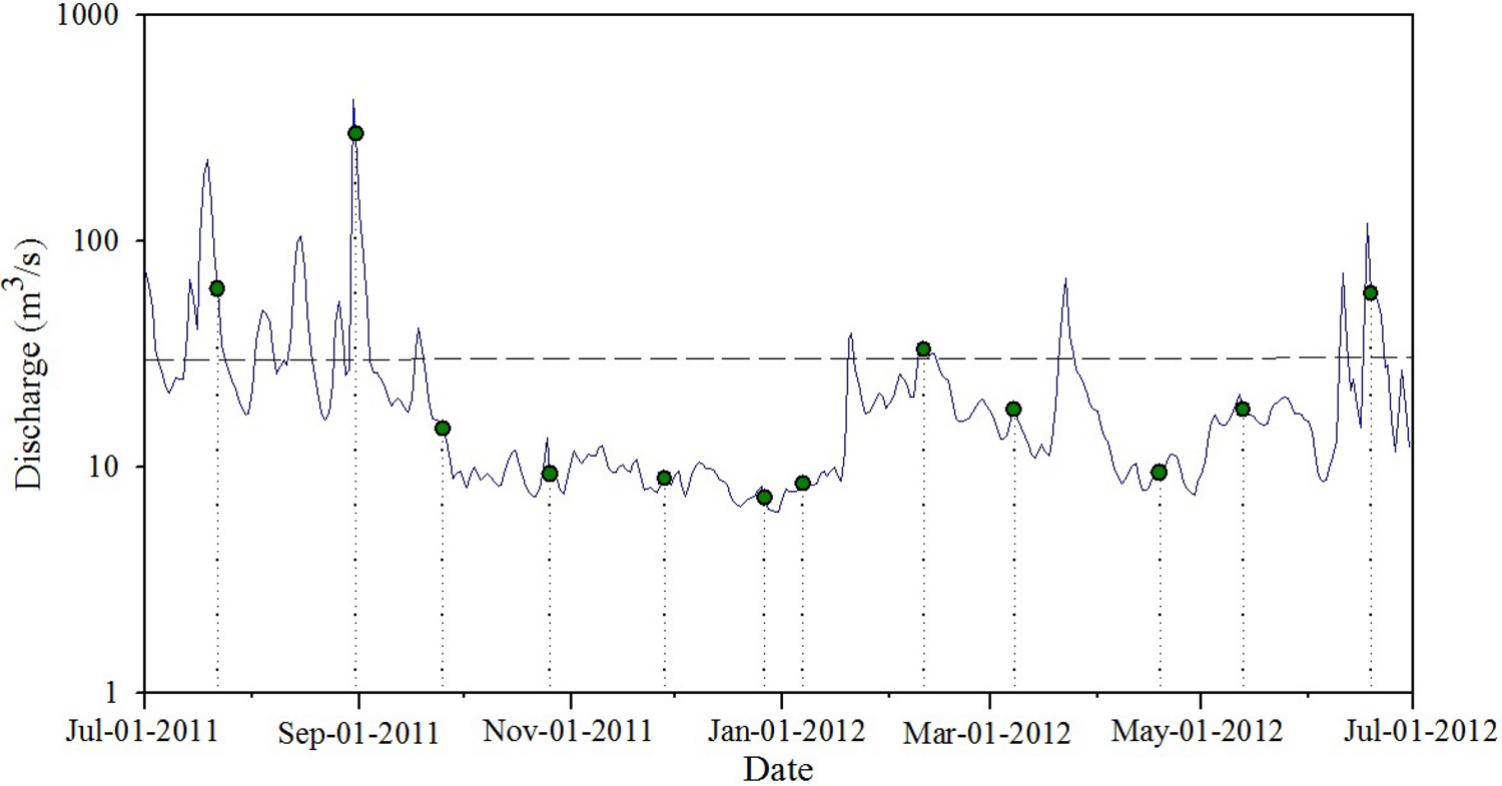
Discharge at the Hengtang Village gauging station from July 2011 to June 2012. The solid line indicates daily discharge. The dashed line indicates the mean daily discharge from a previous record of 23 year of discharge data during 1990–2013. Dots indicates dates when streamwater samples were collected.

Discharge at X1 for the sample collection times was obtained from the Hengtang Village gauging station (middle branch of Xitiao River, Huzhou hydrology station), where discharge is continuously measured as part of the national hydrological monitoring system (Fig. 4). This gauging station on the middle branch of the Xitiao River is free-flowing, and the discharge is strongly correlated with the discharge in the main branches of the river further downstream. Discharges in X2, X4 and X8 were so large that they can’t be obtained by in situ measurement. Therefore, discharge at this gauging station was also used to estimate the discharge at X2, X4 and X8. We used the mean discharge over the 24-h period on each sampling date. Discharge for the other 10 small streams was estimated from measurements of stream velocity and depth collected at regular intervals across the stream on each sampling occasion by a SonTek/YSI-Flow Tracker acoustic current meter and wading rod. A previous record of 23 years of discharge data between 1990 and 2013 was measured with a mean daily discharge of 27.65 m^3^/s (Huzhou Water Resources Bureau, 2014; http://slj.huzhou.gov.cn.). The sampling periods were classified into high and low flow conditions (Fig. 6), based on daily discharge records in the Hengtang Village gauging station using the method of^14^. Low flow conditions existed on 25 September 2011, 26 October 2011, 28 November 2011, 27 December 2011, 7 January 2012, 8 March 2012, 19 April 2012, and 13 May 2012, and high flow conditions existed on 22 July 2011, 31 August 2011, 11 February 2012, and 19 June 2012 (Fig. 6).

### Data analysis

**Figure 7:**
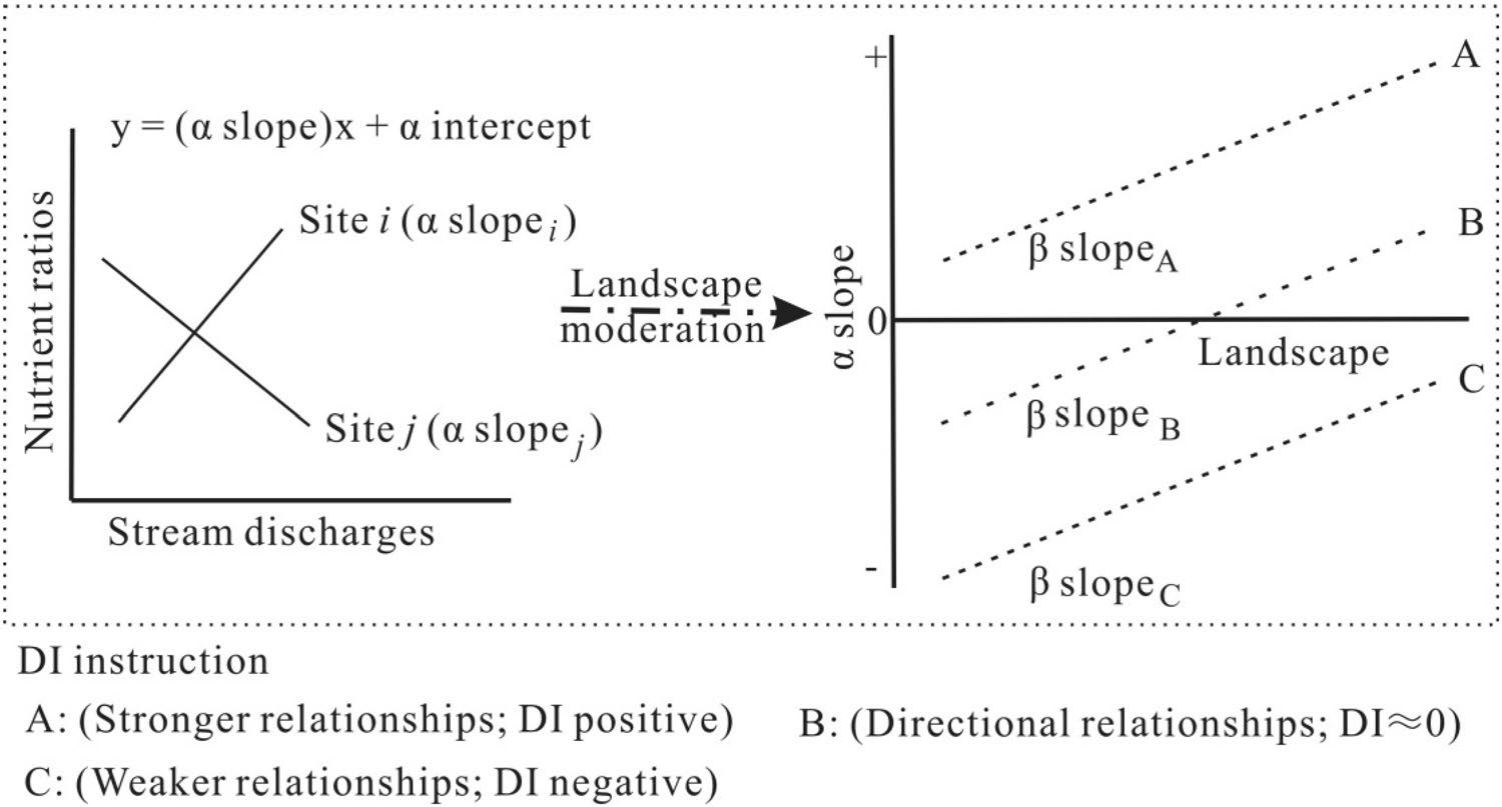
Different relationships between stream discharge and stream water nutrient stoichiometry under different landscape composition.

The k-means clustering method was used for grouping catchments based on nine landscape variables, e.g., mean percentage of slope, stream length, stream density, percentage of cropland, percentage of grassland, percentage of urban, percentage of forest, percentage of bare land, and percentage of wetland. ANOVAs with Tukey’s Post Host Multiple Comparisons for Observed Means were used to examine the relative effects of sampling sites and sampling dates on stream water C:N, C:P, and N:P ratios in the Xitiao River streams during the study period. Linear regressions were used to assess the relationships between stream water C:N, C:P, N:P ratios and discharge among catchment groups and between streamflow conditions. A multistep regression approach (Fig. 7), developed by^16^, was subsequently used to quantify the impacts of catchment properties on the stream-specific relationships between stream water C:N:P ratios and discharge. To calculate the multistep regression, the α slopes were derived from regression analysis of relationships between stream discharge and each nutrient ratio for each sampling site. These were then related to each landscape variable in the 14 sampling streams to generate a set of β slopes, which were mainly used to interpret the impact of each landscape variable on the spatially variable relationships between stream discharge and stream water C:N:P ratios in the Xitiao River. If the β slope differed significantly from zero, three types of relationships (e.g., positive/stronger, negative/weaker or directional relationship; Fig. 7) were determined using a Directional Index (DI) that was calculated by subtracting the absolute value of the predicted α slope at the lowest value of some landscape variable from that predicted at the highest value of the same landscape variable. When the β slope was not significantly different from zero, differences in the landscape components among catchments could not be used to explain the variability in the relationships between the stream discharge and stream water C:N:P ratios. An example of the DI calculation process can be found in the Appendix.

## Supplementary Information

Supplementary Information.
