# Publisher Correction: Catchment landscape components alter relationships between discharge and stream water nutrient ratios in the Xitiao River Basin China

**DOI:** 10.1038/s41598-021-96905-4

**Published:** 2021-08-24

**Authors:** Changjun Gao, Wei Li, Lijuan Cui, Qiongfang Ma, Jian Cai

**Affiliations:** 1 Institute of Wetland Research, Chinese Academy of Forestry, Beijing Key Laboratory of Wetland Services and Restoration, No. 1, Dongxiaofu, Haidian District, Beijing, 100091 People’s Republic of China; 2 Guangdong Provincial Key Laboratory of Silviculture, Protection and Utilization, Guangdong Academy of Forestry, Guangzhou, 510520 People’s Republic of China; 3 Jilin Provincial Academy of Forestry Science, Changchun, 130033 People’s Republic of China

Correction to: *Scientific Reports* 10.1038/s41598-021-89804-1, published online 17 May 2021

The original version of this Article omitted an affiliation for Changjun Gao.

In addition, Wei Li was incorrectly affiliated with ‘Guangdong Provincial Key Laboratory of Silviculture, Protection and Utilization, Guangdong Academy of Forestry, Guangzhou, 510520, People’s Republic of China’. Jian Cai was incorrectly affiliated with ‘Institute of Wetland Research, Chinese Academy of Forestry, Beijing Key Laboratory of Wetland Services and Restoration, No. 1, Dongxiaofu, Haidian District, Beijing, 100091, People’s Republic of China’

The correct affiliations are listed below.

Affiliation 1:

Institute of Wetland Research, Chinese Academy of Forestry, Beijing Key Laboratory of Wetland Services and Restoration, No. 1, Dongxiaofu, Haidian District, Beijing, 100091, People’s Republic of China

Changjun Gao, Wei Li, Lijuan Cui

Affiliation 2:

Guangdong Provincial Key Laboratory of Silviculture, Protection and Utilization, Guangdong Academy of Forestry, Guangzhou, 510520, People’s Republic of China

Changjun Gao & Jian Cai

Affiliation 3:

Jilin Provincial Academy of Forestry Science, Changchun, 130033, People’s Republic of China

Qiongfang Ma

The original Article has been corrected.

