# Supplementary material for: Catchment landscape components alter relationships between discharge and stream water nutrient ratios in the Xitiao River Basin China
Source: Sci Rep. 2021 May 17;11:10466. doi: 10.1038/s41598-021-89804-1 (PMC8128907; doi:10.1038/s41598-021-89804-1)
Supplement: Supplementary file 1 — Supplementary Information. [file 41598_2021_89804_MOESM1_ESM.docx]

**Appendix A. Supplementary data**

Catchment landscape components alter relationships between discharge and stream water nutrient ratios in the Xitiao River Basin China

Changjun Gao • Wei Li • Lijuan Cui* • Qiongfang Ma • Jian Cai

C. J. Gao

Postal address: ^a^ Institute of Wetland Research, Chinese Academy of Forestry, Beijing Key Laboratory of Wetland Services and Restoration, Beijing, 100091, P.R. China; ^b^ Guangdong Academy of Forestry, Guangdong Provincial Key Laboratory of Silviculture, Protection and Utilization, Guangzhou 510520, P.R. China

Email: gaochangjun015@163.com

L. J. Cui

Postal address: ^a^ Institute of Wetland Research, Chinese Academy of Forestry, Beijing Key Laboratory of Wetland Services and Restoration, No. 1, Dongxiaofu, Haidian District, Beijing, 100091, P. R. China

E-mail: wetlands108@126.com, lkyclj@126.com;

W. Li

Postal address: ^a^ Institute of Wetland Research, Chinese Academy of Forestry, Beijing Key Laboratory of Wetland Services and Restoration, Beijing, 100091, P.R. China

Email: wetlands207@163.com

Q. F. Ma

Postal address: ^c^ Jilin Provincial Academy of Forestry Science, Changchun, 130033, P.R. China

Email: youzi841128@qq.com

J. Cai

Postal address: ^b^ Guangdong Academy of Forestry, Guangdong Provincial Key Laboratory of Silviculture, Protection and Utilization, Guangzhou 510520, P.R. China

Email: caijian@sinogaf.cn

*Corresponding author:

Lijuan Cui

Postal address: ^a^ Institute of Wetland Research, Chinese Academy of Forestry, Beijing Key Laboratory of Wetland Services and Restoration, No. 1, Dongxiaofu, Haidian District, Beijing, 100091, P. R. China

E-mail: wetlands108@126.com, lkyclj@126.com; Tel: 86-010-62824151; Fox mail: 86-010-62824151

**Table A.1. Correlation coefficients among catchment properties from the XRB streams**

| Property | Slope | Length | Density | %crop | %grass | %urban | %forest | %bare | %wet |
| --- | --- | --- | --- | --- | --- | --- | --- | --- | --- |
| Slope | 1.00 | 0.36 | –0.39 | –**0.93** | –0.45 | –0.22 | **0.95** | –**0.54** | –**0.65** |
| Length |  | 1.00 | –0.17 | –0.17 | –0.06 | –0.23 | 0.21 | 0.17 | 0.22 |
| Density |  |  | 1.00 | 0.29 | 0.45 | 0.33 | –0.43 | 0.49 | 0.24 |
| %crop |  |  |  | 1.00 | 0.44 | 0.00 | –**0.94** | **0.71** | **0.71** |
| %grass |  |  |  |  | 1.00 | 0.20 | –**0.58** | **0.60** | **0.69** |
| %urban |  |  |  |  |  | 1.00 | –0.32 | –0.16 | 0.09 |
| %forest |  |  |  |  |  |  | 1.00 | –**0.67** | –**0.76** |
| %bare |  |  |  |  |  |  |  | 1.00 | **0.66** |
| %wet |  |  |  |  |  |  |  |  | 1.00 |

**
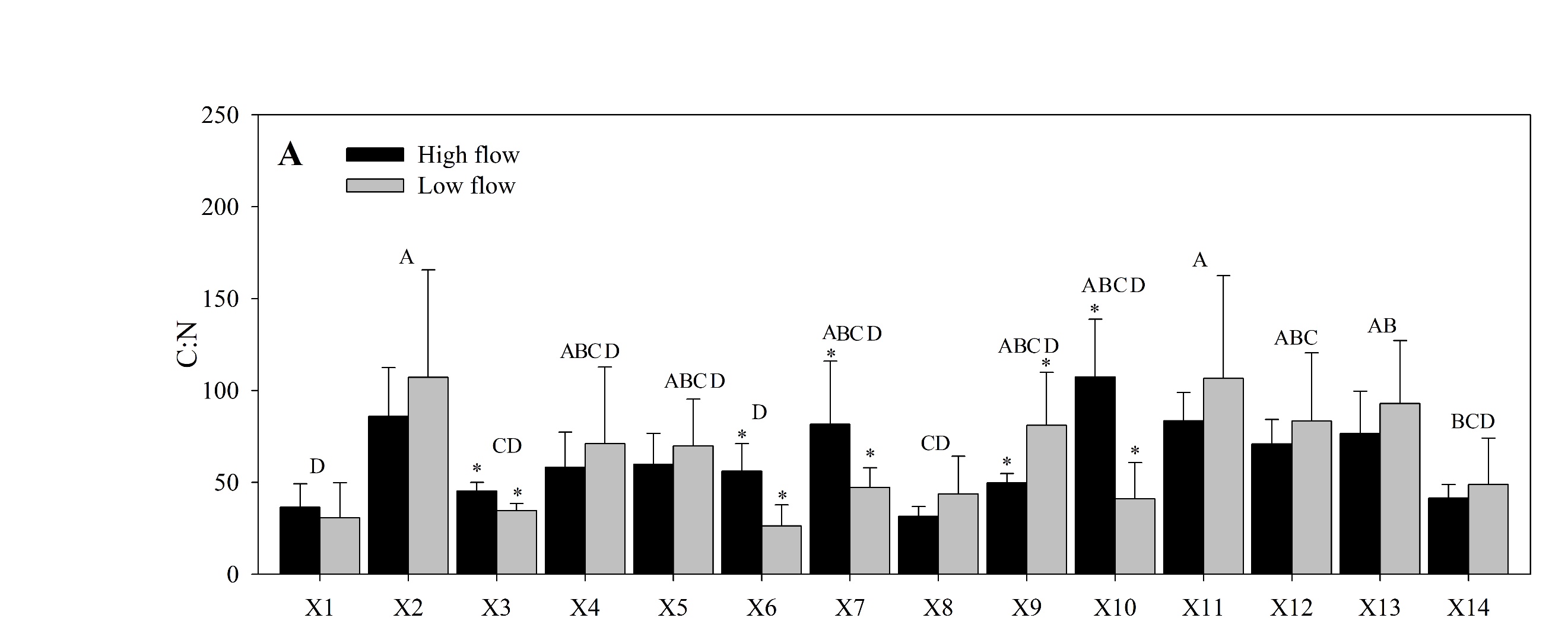

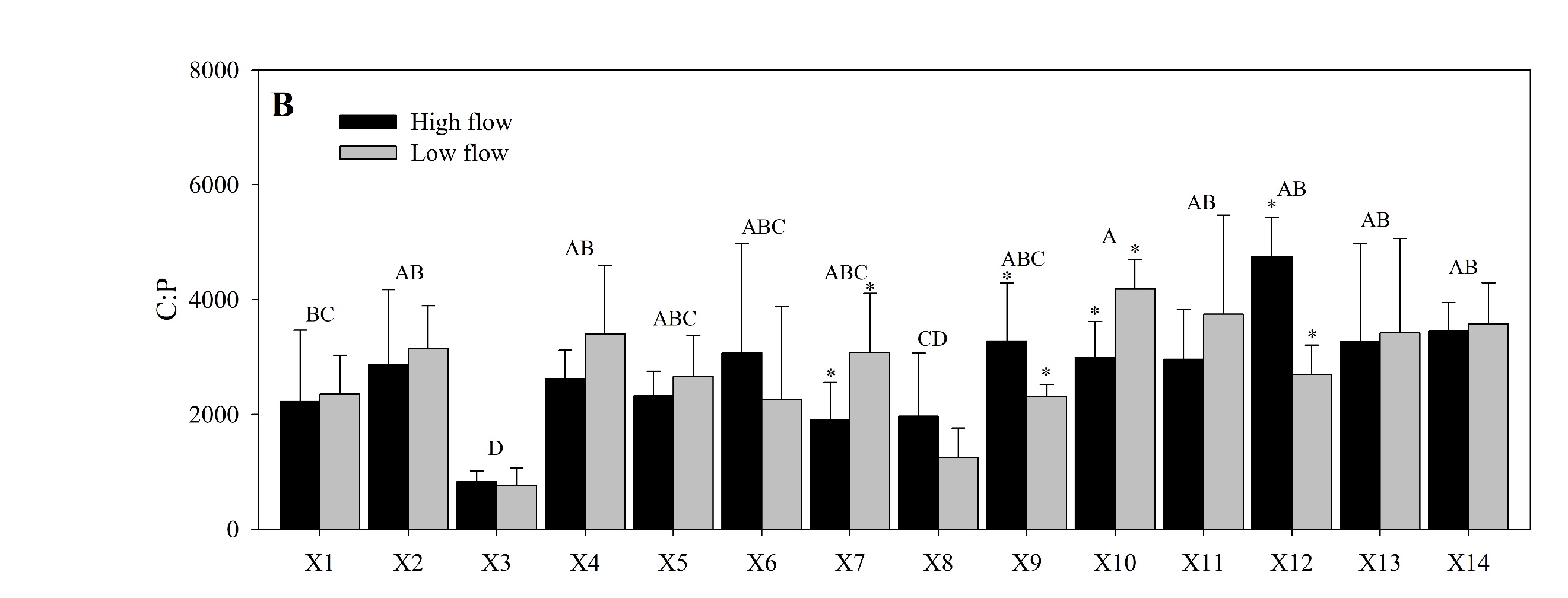

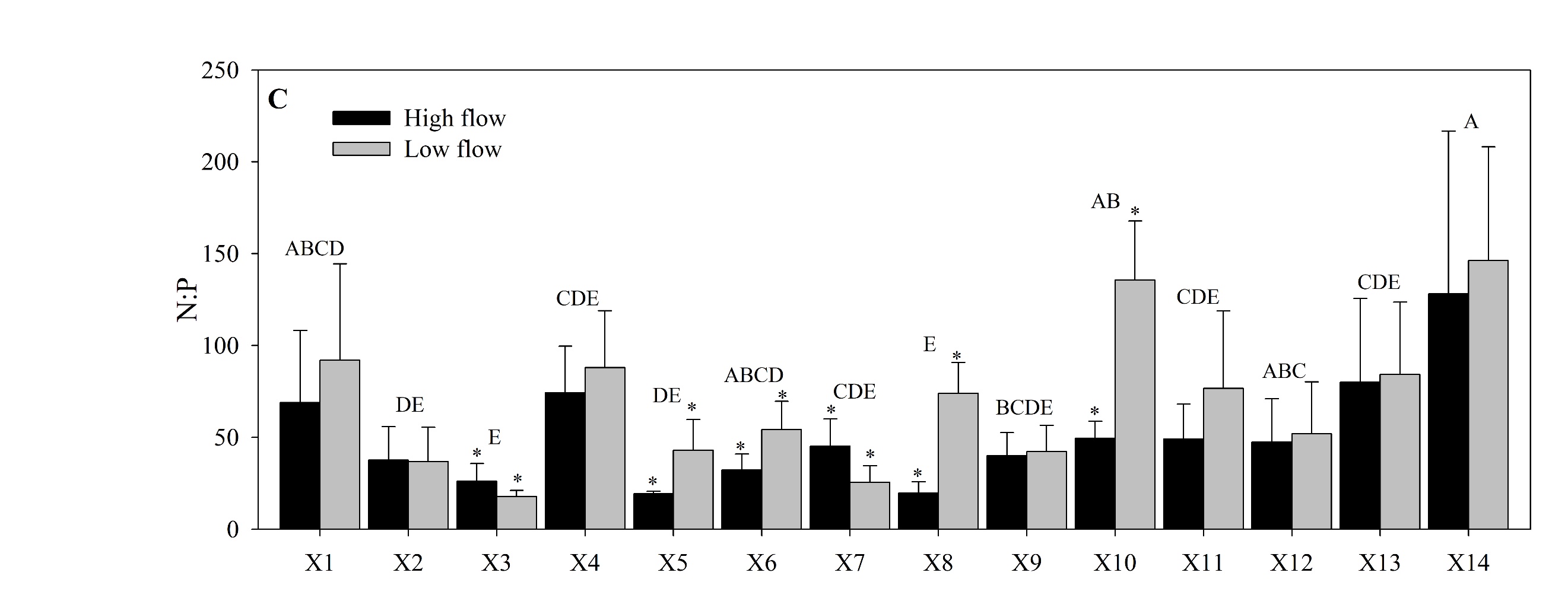
**

Figure A.1. C:N:P ratios in fourteen sampling streams during periods of high and low flow

Note: Asterisk means significant difference (*p* < 0.05) for each sampling stream in different flow conditions. Alphabet means significant difference (*p* < 0.05) among different sampling stream sites during the whole flow periods.

**An example of the Directional Index (DI) calculation process**

As indicated in the “Data analysis” section in the main paper, the DI calculation process can be classified into three steps. Firstly, the α slopes were derived from regression analysis of relationships between river discharge and each nutrient ratio for each sampling site. Taking the C:P ratios as an example, there were monthly C:P ratio and stream discharge data in each of the fourteen sampling streams from July 2011 to June 2012. So, there was a unique regression relationship (e.g., slope, R^2^, *P*-value,) for each of the sampling streams (Figure A2). In Figure A2, these slopes mean “α slope”. Secondly, the “α slope” was then related to landscape variables in the fourteen sampling catchments. So, there was another unique regression relationship for the α slope and landscape property (e.g., slope, R^2^, *P*-value; see in Figure A3). In Figure A3, these slopes mean “β slopes”. Finally, DI was calculated based on two scenarios: 1) The α--landscape regression relationship was not statistically significant. For example, the *P-*value was greater than or equal to 0.05 in Figure A3 and Figure 3b (e.g., stream length, stream density, percentage of grassland, percentage of urban and percentage of bare land). This means that there was no DI. Or, in other words, changes in the five landscape properties (e.g., stream length, stream density, percentage of grassland, percentage of urban and percentage of bare land) among catchments had no effects on the relationship between the C:P ratio and river discharge in the study region. 2) The α--landscape regression relationship was statistically significant. For example, *P* value was less than 0.05 in Figure A3 (e.g., mean percentage of slope, percentage of cropland, percentage of forest and percentage of wetland). Then, DI was calculated based on equation (1) as follows:

$DI=\left| \alpha slope \right|_{land(l)}-\left| \alpha slope \right|_{land(s)}$ (1)

Where *land (l)* is the largest value of some landscape variable (e.g., mean percentage of slope, percentage of cropland, percentage of forest and percentage of wetland) among the catchments in the Xitiao River basin; *land (s)* is the smallest value of some landscape variable among catchments in the Xitiao River basin; |α slope |*_land(l)_* means α absolute value when the independent variable is largest in the regression model in Figure A3. |α slope |*_land(s)_* means α absolute value when independent variable is smallest in the regression model in Figure A3. As shown in Figure 7, if DI ≈ 0 (Figure 7 B), this indicates directional relationships. If DI > 0 (Figure 7 A), this means stronger relationships. If DI < 0 (Figure 7 C), this means weaker relationships. Detailed information can be found in Table A2.

**Table A.2. DI calculation process based on landscape variable and α slope**

| **Landscape type** | **Landscape variable value** | | **α slope** | **DI** | **DI instruction** |
| --- | --- | --- | --- | --- | --- |
| Mean percentage of slope | Largest value | 1.4428 | -0.113 | -0.002 | DI ≈ 0;  Directional relationships |
|  | Smallest value | 0.4133 | 0.115 |  |  |
| Percentage of cropland | Largest value | 0.9630 | 0.086 | -0.024 | DI ≈ 0;  Directional relationships |
|  | Smallest value | 0.2299 | -0.110 |  |  |
| Percentage of forest | Largest value | 1.2844 | -0.139 | 0.038 | DI ≈ 0;  Directional relationships |
|  | Smallest value | 0.4655 | 0.101 |  |  |
| Percentage of wetland | Largest value | 0.2220 | 0.096 | -0.072 | DI ≈ 0;  Directional relationships |
|  | Smallest value | 0.0165 | -0.168 |  |  |

Explanation for Table A2:

If α slope was greater than zero in the largest and smallest value of a landscape variable, then DI was significantly larger than zero (Figure 7 A). This means stronger (positive) relationships. In other words, the change of landscape properties would strengthen the correlation of C:P ratio and river discharge.

If α slope was less than zero in the largest and smallest value of a landscape variable, then DI was significantly lower than zero (Figure 7 C). This means weaker (negative) relationships. In other words, the change of landscape properties would weaken the correlation of C:P ratio and river discharge.

If α slope was greater than zero in the largest value of landscape variable and was less than zero in smallest value of a landscape variable, and vice versa, then DI approximately equaled to zero (Figure 7 B). This means directional relationships. In other words, the change of landscape properties would not strengthen or weaken the correlation of C:P ratio and river discharge but could change the direction of correlation of the C:P ratio and river discharge. e.g., from α = −*a* (landscape variable) + *b* to α = *a* (landscape variable) + *b*; *a* value changed from positive to negative or from negative to positive. As shown in Figure A3 and Table A2, all the five landscape variables have effects on altering the direction of the C:P-discharge relationship.


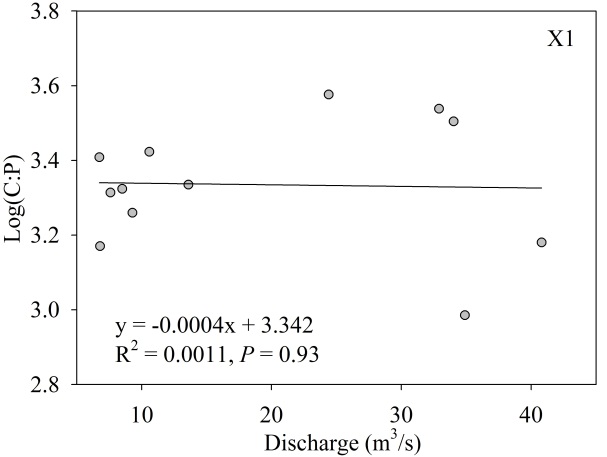

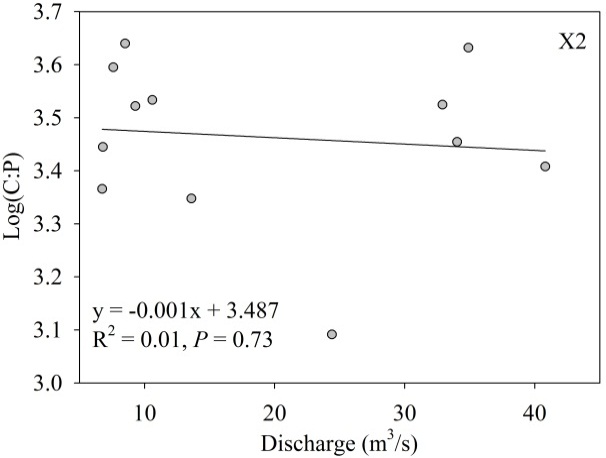

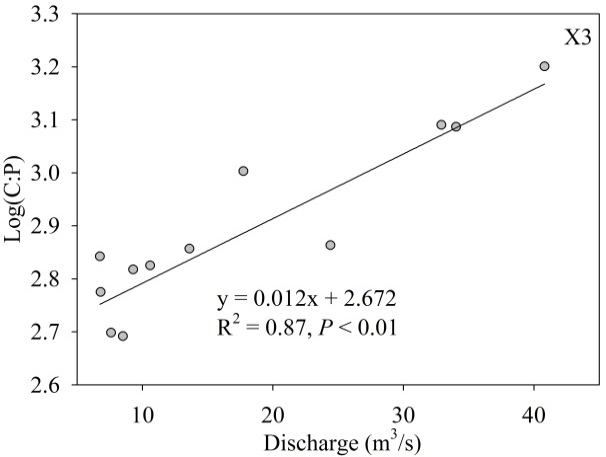

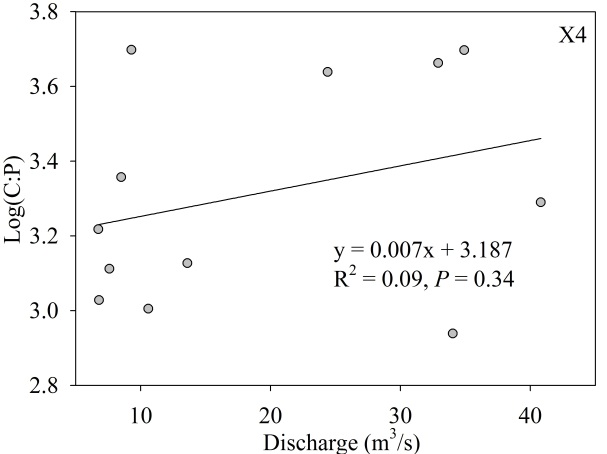

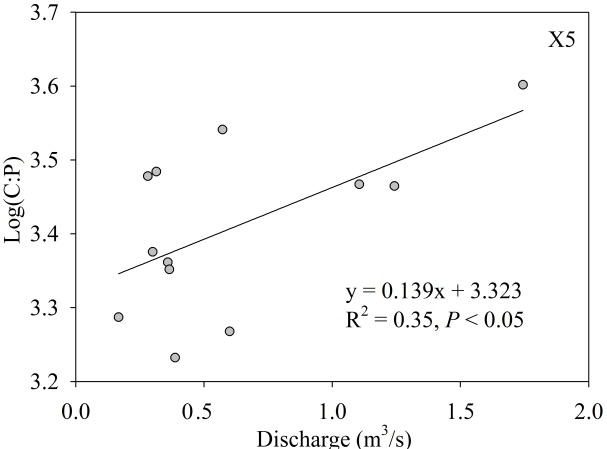

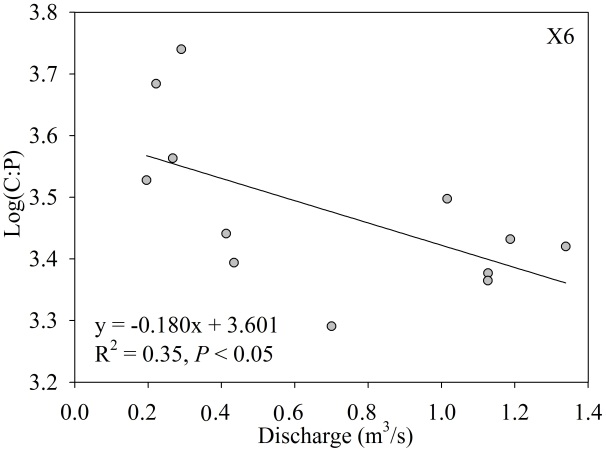

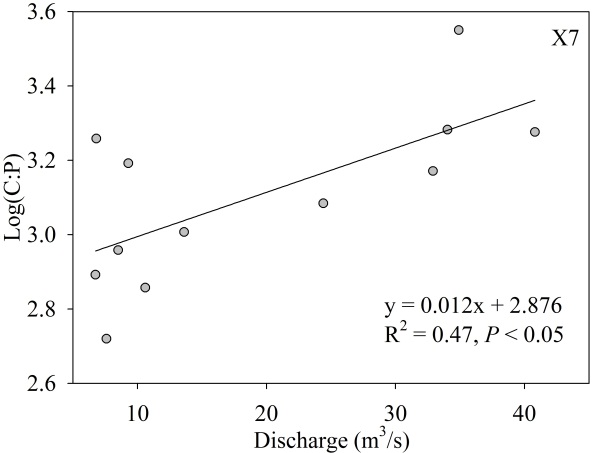

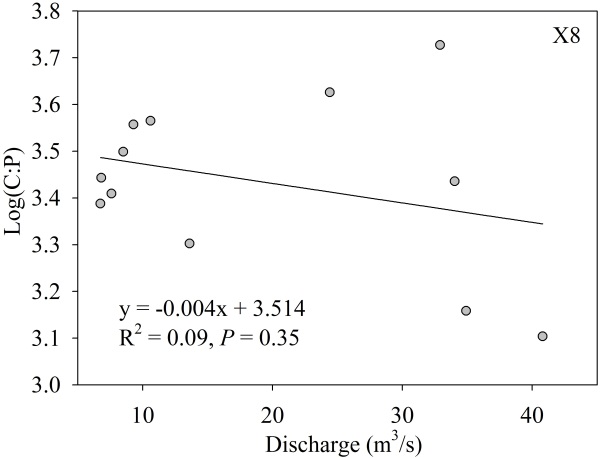

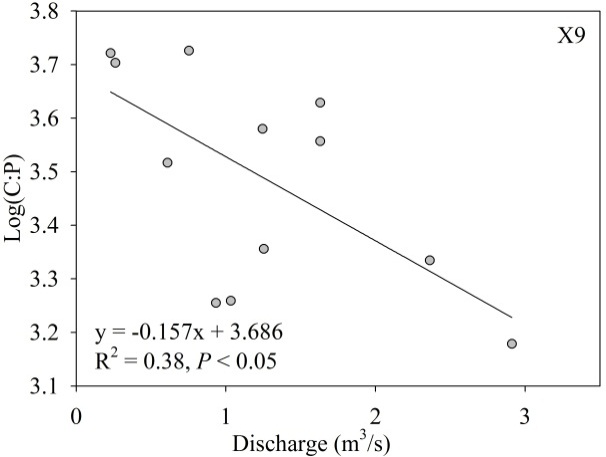

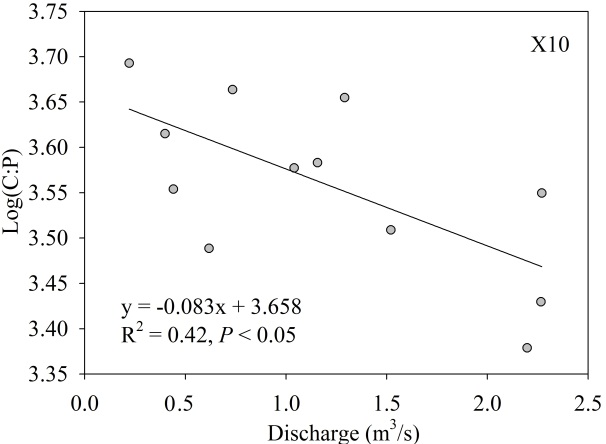

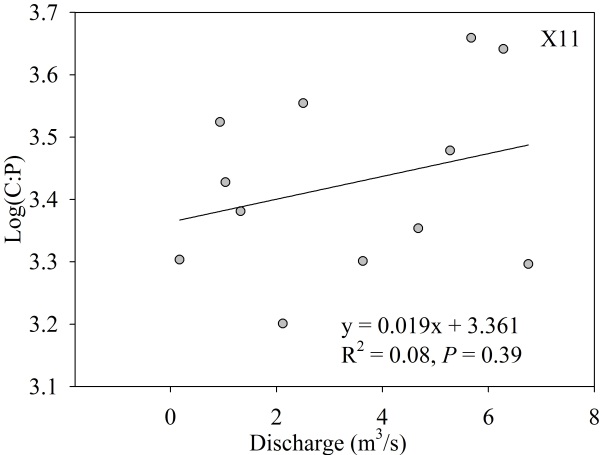

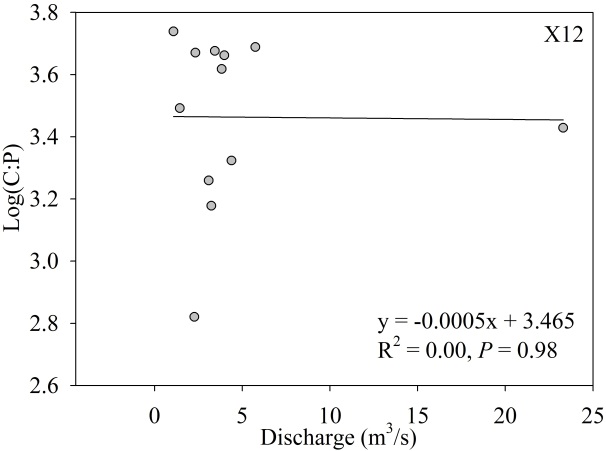

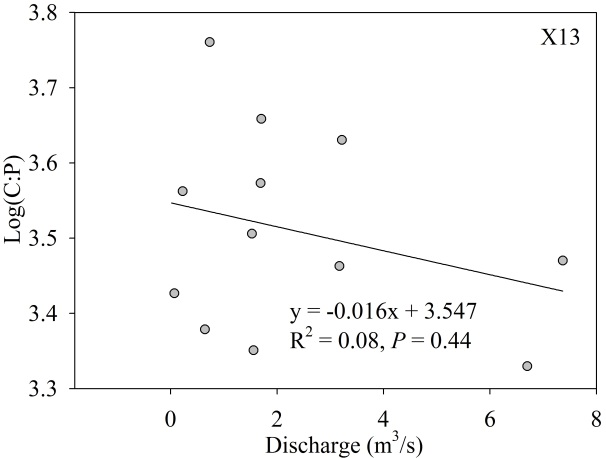

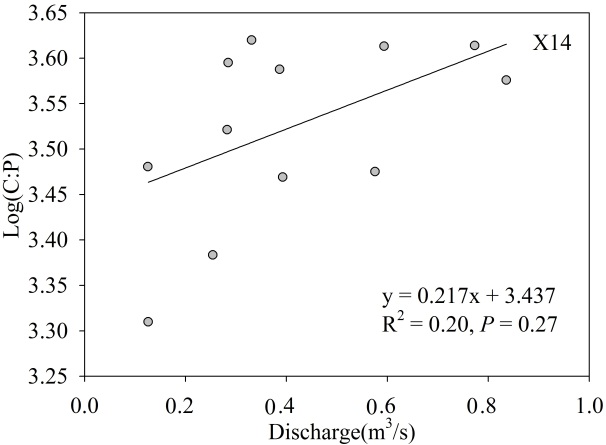


Figure A.2. Relationships between C:P ratios and discharges in streams from X1 to X14


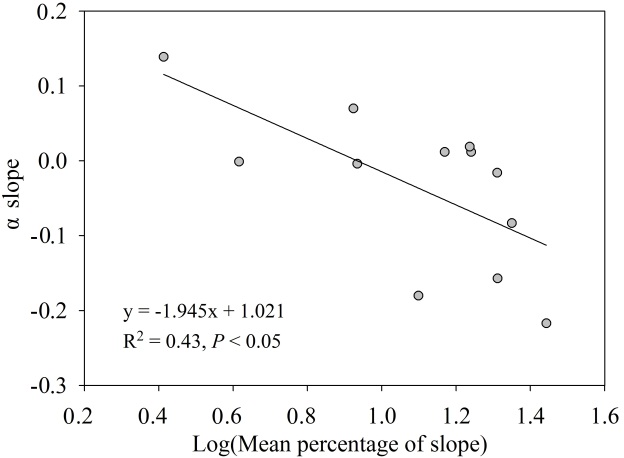

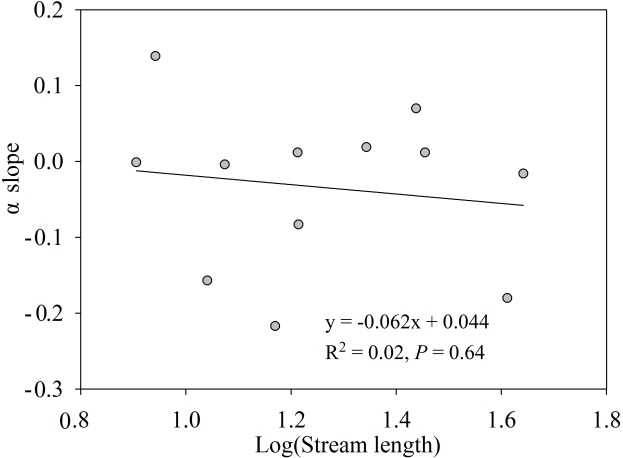

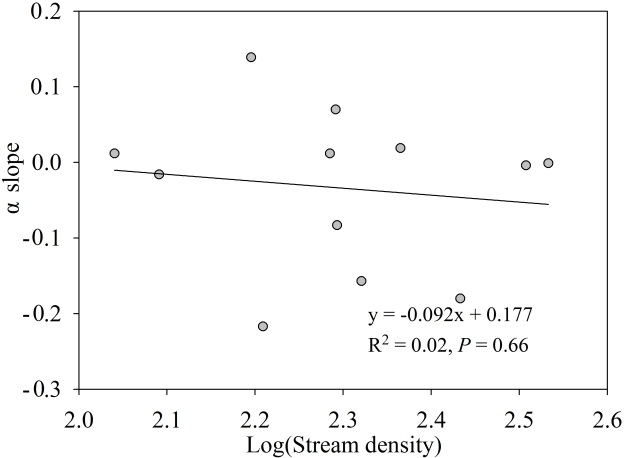

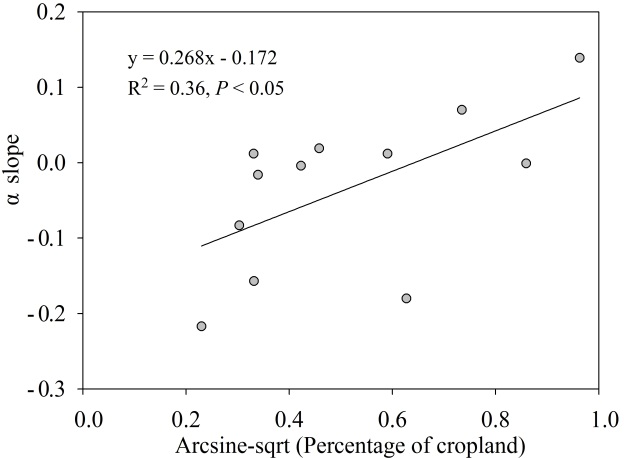

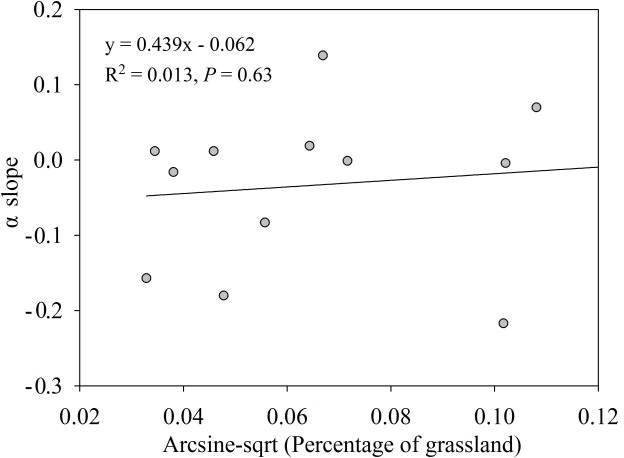

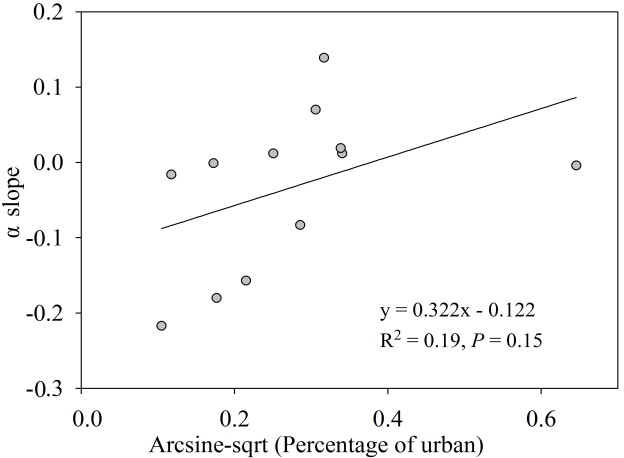

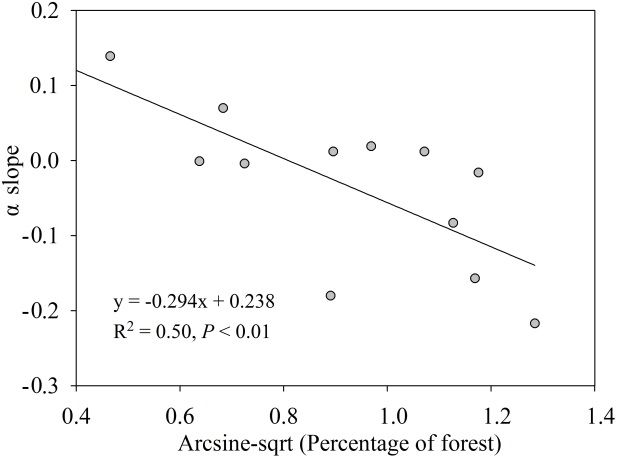

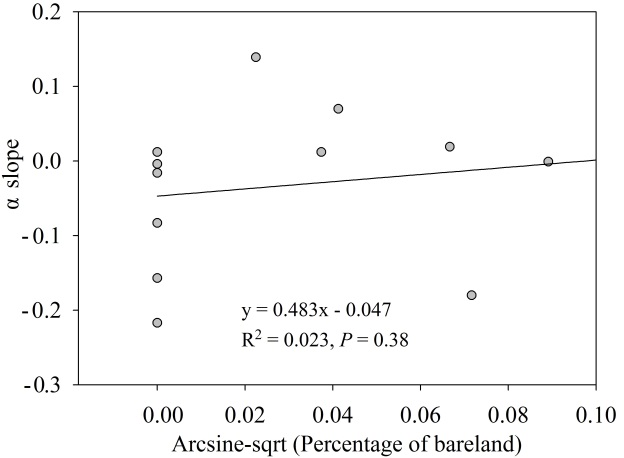

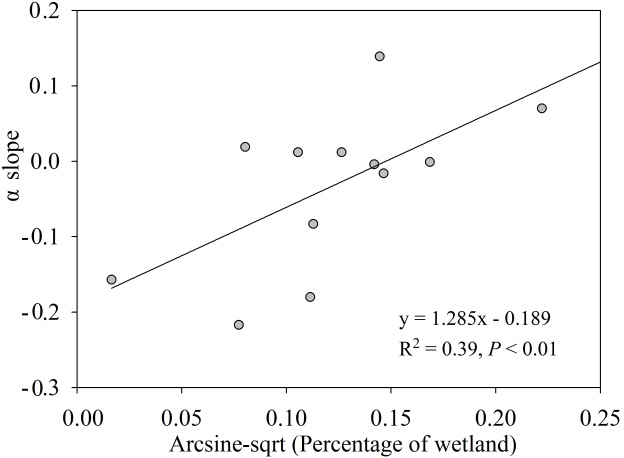


Figure A.3. Relationships between α slope and landscape property in fourteen streams
